# p75^NTR^-dependent activation of NF-κB regulates microRNA-503 transcription and pericyte–endothelial crosstalk in diabetes after limb ischaemia

**DOI:** 10.1038/ncomms9024

**Published:** 2015-08-13

**Authors:** Andrea Caporali, Marco Meloni, Audrey Nailor, Tijana Mitić, Saran Shantikumar, Federica Riu, Graciela B. Sala-Newby, Lorraine Rose, Marie Besnier, Rajesh Katare, Christine Voellenkle, Paul Verkade, Fabio Martelli, Paolo Madeddu, Costanza Emanueli

**Affiliations:** 1 School of Clinical Sciences, Bristol Heart Institute, Bristol BS2 8HW, UK; 2 University/British Heart Foundation Centre for Cardiovascular Science, The Queen's Medical Research Institute, University of Edinburgh, Edinburgh EH16 4TJ, UK; 3 Molecular Cardiology Laboratory, IRCCS-Policlinico San Donato, Milan 20097, Italy; 4 Wolfson Bioimaging Facility, University of Bristol, Bristol BS2 8HW, UK; 5 National Institute of Heart and Lung, Imperial College of London, London SW7 2AZ, UK

## Abstract

The communication between vascular endothelial cells (ECs) and pericytes in the microvasculature is fundamental for vascular growth and homeostasis; however, these processes are disrupted by diabetes. Here we show that modulation of p75^NTR^ expression in ECs exposed to high glucose activates transcription of miR-503, which negatively affects pericyte function. p75^NTR^ activates NF-κB to bind the miR-503 promoter and upregulate miR-503 expression in ECs. NF-κB further induces activation of Rho kinase and shedding of endothelial microparticles carrying miR-503, which transfer miR-503 from ECs to vascular pericytes. The integrin-mediated uptake of miR-503 in the recipient pericytes reduces expression of *EFNB2* and *VEGFA*, resulting in impaired migration and proliferation. We confirm operation of the above mechanisms in mouse models of diabetes, in which EC-derived miR-503 reduces pericyte coverage of capillaries, increased permeability and impaired post-ischaemic angiogenesis in limb muscles. Collectively, our data demonstrate that miR-503 regulates pericyte–endothelial crosstalk in microvascular diabetic complications.

## Abstract (web summary)

Vascular function and repair is impaired in patients with diabetes. Here, Caporali *et al.* report that activation of the neurotrophin receptor in vascular endothelial cells induces the antiangiogenic miR-503, which impairs the function of neighbouring pericytes upon microparticle-mediated transfer.

## Graphical abstract

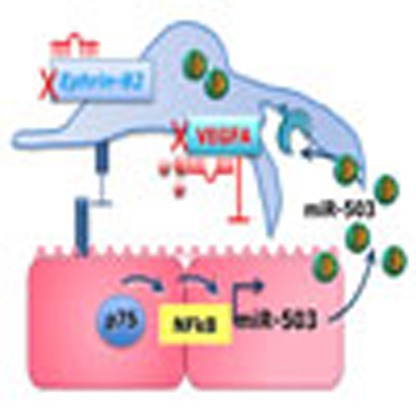

Hyperglycaemia causes vascular endothelial cell (EC) dysfunction, microvascular rarefaction and macrovascular disease during diabetes^1^. Peripheral vascular complications, including non-healing skin ulcers, frequently have deleterious outcomes resulting in foot or lower limb amputation in diabetic patients. This in turn is associated with the high rates of morbidity and mortality, at a tremendous cost to the national health care^2^. The evolution of ischaemic disease in diabetic patients is worsened because of impairment of the reparative angiogenesis process^3^. ECs represent major sites of hyperglycaemic damage due to their ability to readily accumulate D-glucose. Thus, altered intracellular signalling under diabetes induces a phenotypic switch from the normal quiescent profile to a more apoptotic, pro-inflammatory or antiangiogenic phenotype of ECs^4^. The dysfunction, degeneration and loss of vascular pericytes in diabetes additionally contribute to microvascular complications in the retina^5^, kidney^6^ and skeletal muscles^7^. Yet, it still remains a challenge to define the master regulators of gene expression in the vasculature during diabetes. Nevertheless, an emerging picture is already developing, whereby microRNAs (miRNAs) are deemed to be critical regulators of this process. MiRNAs are small non-coding RNAs of ∼22 nucleotides, which mediate the degradation of target mRNAs or their translational arrest through base pairing with the target gene 3′-untranslated regions (UTR)^8^. The characterization of miRNAs in vascular cells has opened novel therapeutic options for the prevention of vascular and cardiovascular diseases (reviewed in ref. 9). We have previously revealed the significance of miR-503 in diabetes-associated endothelial dysfunction and impaired post-ischaemic vascular repair^10^. The expression of miR-503 is upregulated in cultured ECs under conditions mimicking hyperglycaemia and ischaemia. Moreover, miR-503 expression is upregulated in the ischaemic limb muscles of diabetic mice and patients. Further, antagonizing miR-503 in mice improved the post-ischaemic reparative neovascularization and restores the expression of target genes in the diabetic and ischaemic limb muscles^10^. However, the transcriptional regulation of miR-503 by diabetes and ischaemia has not been investigated yet. Similarly to miR-503, we have also identified that the p75 neurotrophin receptor (p75^NTR^) is induced in microvascular ECs in association with diabetes and limb ischaemia, and that it impairs angiogenesis *in vitro* and *in vivo*^11^,^12^. The p75^NTR^ is a multifunctional membrane receptor belonging to the tumour-necrosis factor receptor superfamily, originally identified as a receptor for nerve growth factor^13^. The function of p75^NTR^ varies considerably depending on the cellular milieu in which this receptor is expressed. P75^NTR^ can be activated in both a ligand-dependent and -independent manner; therefore, its signalling proceeds via recruitment and release of the cytoplasmic effectors to and from its intracellular domain. The activation of p75^NTR^ can lead to activation of three main pathways: NF-κB pathway, apoptotic signalling through caspase activation and via the small GTPase RhoA^13^.

Of further relevance for this study, we have previously shown that increased p75^NTR^ expression increases shedding of microparticles (MPs) from the cultured ECs^11^. Recent studies have also identified that miRNAs are released from cells into different types of extracellular vesicles (EVs), such as exosomes (30–100 nm) or MPs (100 nm–1 μm), which circulate within the peripheral blood^14^,^15^,^16^. The circulating EVs have been proposed to serve as the signalling molecules and mediators of the intercellular communication^17^. In line with this hypothesis, EVs released from cells are able to alter gene expression and functional behaviour in recipient cells by transferring their miRNA content^14^,^15^,^16^.

ECs and pericytes establish a cellular crosstalk in the microvasculature, which is fundamental for the regulation of angiogenesis, microvascular stabilization and permeability, but is disrupted under diabetes^18^. However, it has not yet been demonstrated whether the trafficking of miRNAs in the MPs from diabetic ECs can influence proximate cells, and in particular neighbouring vascular pericytes.

Here we provide evidence that upregulation of miR-503 through p75^NTR^-dependent activation of NF-κB in ECs impairs endothelial function and angiogenesis. Moreover, we demonstrate that the antiangiogenic properties of miR-503 are conferred by the production and transfer of endothelial MPs carrying miR-503 into recipient pericytes, where miR-503 further suppresses the endothelial–pericyte crosstalk by targeting *EFNB2* and *VEGFA.* The passage of miR-503 from ECs to pericytes, where miR-503 is expressed in lower amount compared with ECs and is not transcribed or processed under diabetes and ischaemia, yet it is in part regulated by β3-integrin antagonists. Moreover, we found that transfer of miR-503 occurs also *in vivo* from ECs to pericytes and it increases vascular permeability during diabetic ischaemia.

## Results

### The p75^NTR^ receptor regulates miR-503 expression

**Figure 1:**
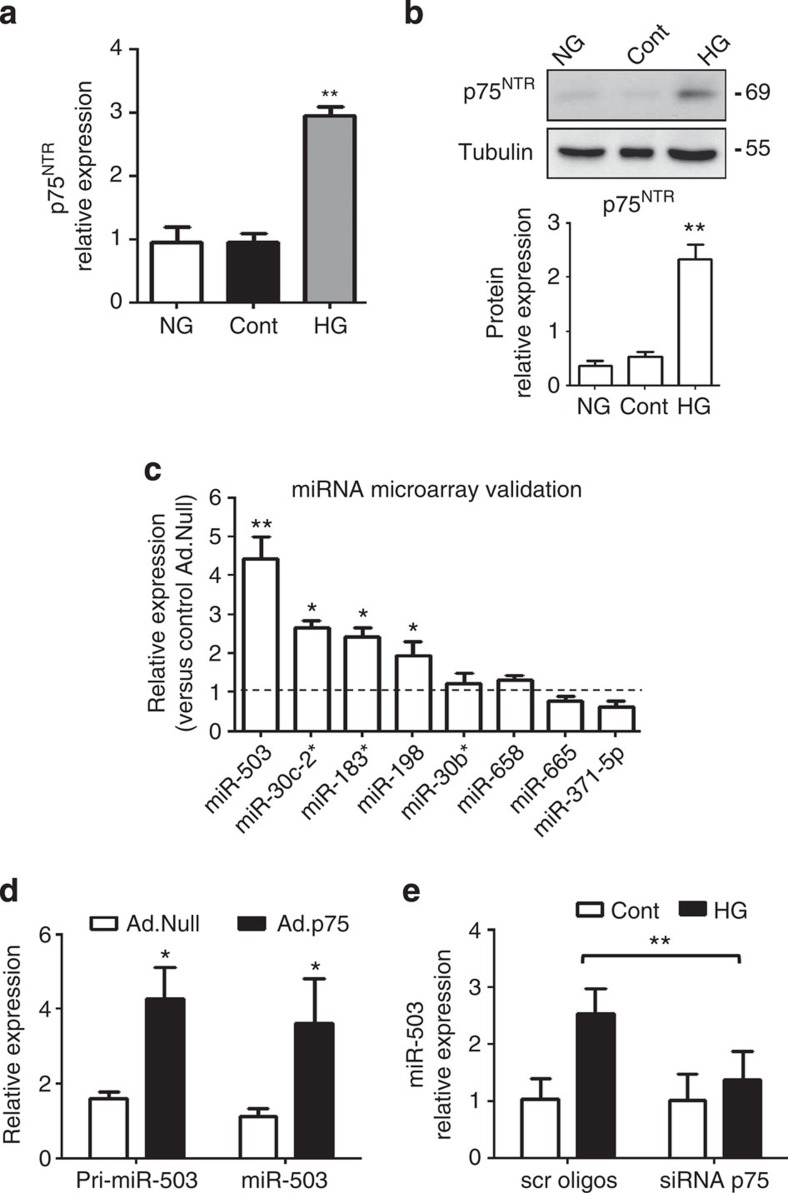
p75^NTR^ regulates miR-503 expression. (**a**) HUVECs were exposed to high glucose (HG), cultured in normal glucose (NG) or osmotic control (Cont; L-Glucose) conditions for 24 h, and p75^NTR^ expression was analysed using qPCR. **P<0.01 versus NG or Cont (*n*=3). (**b**) Representative western blot images and protein quantification of p75^NTR^ and Tubulin expression in total cell extracts. **P<0.01 versus NG or Cont (*n*=3). (**c**) Microarray data validation using qPCR. HUVECs were transduced with *Ad.Null* or *Ad.p75* and qPCR was carried out to measure the expression of top-ranked miRNAs. (**d**) Expression of precursor and mature miR-503 for **c**,**d**; *P<0.05, **P<0.01 versus *Ad.Null* (*n*=3). (**e**) Relative expression of miR-503 in HUVECs transfected with scrambled oligos (scr oligos) or short interfering RNA (siRNA)-p75 oligos and then cultured in Cont or HG for 24 h. **P<0.01 (*n*=5). Unpaired two-tailed Student’s *t*-test or Mann–Whitney nonparametric test was applied. Differences among groups were analysed using two-way analysis of variance followed by Bonferroni *post hoc* test. All values are mean±s.e.m. of three independent experiments.

We have previously reported minimal or no expression of p75^NTR^ in cultured human umbilical vein ECs (HUVECs), human microvascular ECs (HMVECs) under basal conditions and in the capillaries of limb muscles of healthy mice^11^,^12^. In diabetes, especially when associated with tissue ischaemia or injury, the expression of p75^NTR^ increases in the microvascular ECs^11^,^12^ (Supplementary Fig. 1). Exposure of cells to high D-glucose concentrations (HG) and culture in low-growth-factor medium, conditions that mimic diabetes and ischaemia *in vitro* (L-Glucose as an osmotic control; Cont), increases *p75*^NTR^ mRNA and protein expression in both HUVECs (Fig. 1a,b) and HMVECs (Supplementary Fig. 2A,B). The p75^NTR^ expression is not regulated by the osmotic pressure (normal glucose (NG) versus Cont, Fig. 1a,b and Supplementary Fig. 2A,B).

To explore which miRNAs are regulated by p75^NTR^ in ECs, a miRNA microarray was performed in HUVECs overexpressing *p75*^*NTR*^ (adenoviral vector *p75*^*NTR*^: *Ad.p75*; control: *Ad.Null*; Supplementary Fig. 2C) and the top-ranked miRNAs (Supplementary Fig. 2D) were validated using qPCR. This resulted in an increased expression of miR-503, miR-30-c2*, miR-183* and miR-198, with miR-503 being the most upregulated (Fig. 1c). Overexpression of p75^NTR^ further induced the expression of miR-503 precursor (pri-miR-503; Fig. 1d). In addition, the expression of miR-503 increased in HUVECs exposed to HG, with this response being prevented on knockdown of *p75*^*NTR*^ by short interfering RNA (Fig. 1e and Supplementary Fig. 3A,B).

To better clarify the link between miR-503 and p75^NTR^, we analysed the functional effect of miR-503 inhibition in the *p75*^*NTR*^-overexpressing HUVECs. Inhibition of miR-503 was achieved by using adenovirus-*decoy.miR-503* (*Ad.decoy503*) vector, as before^10^. In the p75^NTR^-transduced ECs the proliferation capacities and network formation on Matrigel were impaired^11^, yet these defects could be corrected on addition of *Ad.decoy503* (Supplementary Fig. 3C,D, respectively).

### *In vivo* regulation of miR-503 by p75^NTR^

**Figure 2:**
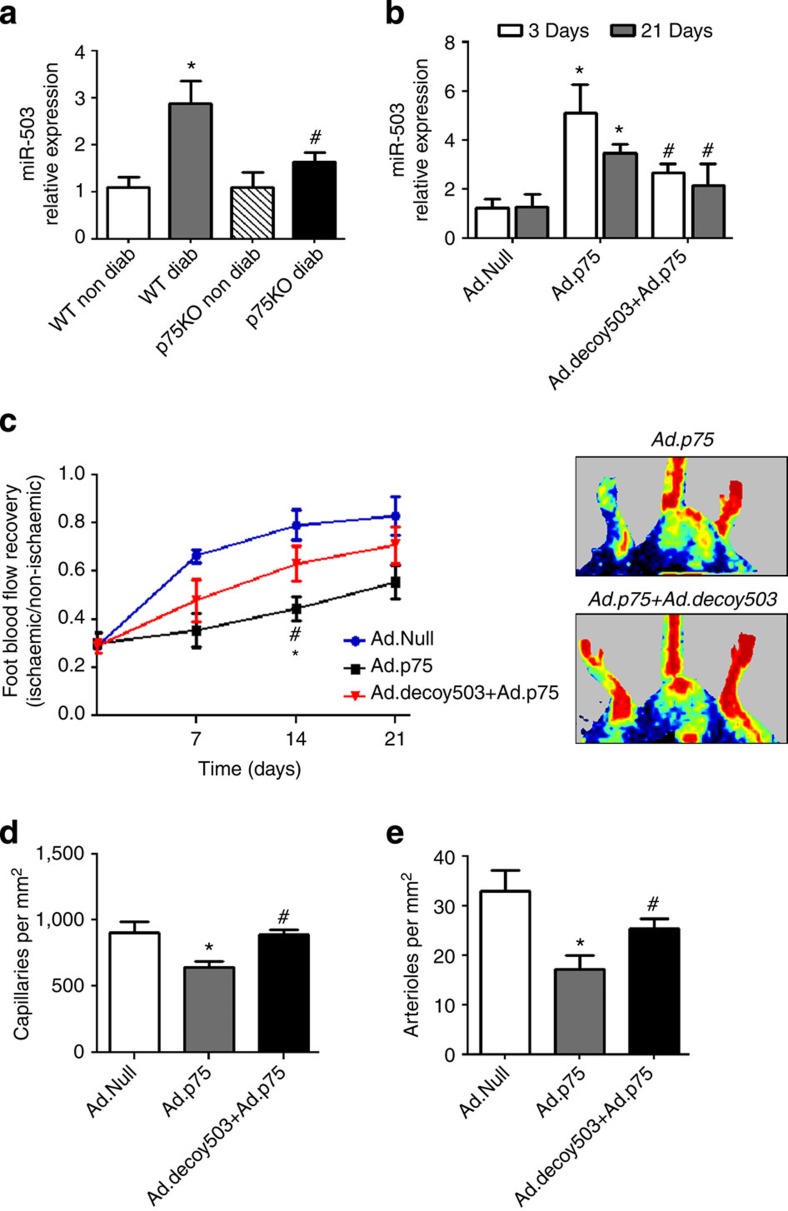
*In vivo* regulation of miR-503 by p75^NTR^. (**a**) Relative expression of miR-503 in ischaemic muscle (3 days post ischaemia) of diabetic and non-diabetic WT and p75KO mice (*n*=6 per group); *P<0.05 versus WT Non Diab; ^#^*P*<0.05 versus WT Diab. (**b**) Relative expression of miR-503 in ischaemic adductors of non-diabetic mice injected with *Ad.p75*, *Ad.Null* or *Ad.p75* and *Ad.decoy503* together (*n*=6 per group). Ad.*Null* was also given to singly injected mice to equalize the virus quantity. (**c**) Line graph shows the time course of post-ischaemic foot blood flow recovery in mice (calculated as the ratio between ischaemic and contralateral foot blood flow; *n*=12 per group). Representative colour laser Doppler images are taken at 14 days post ischaemia. (**d**,**e**) Column graphs show capillary and small arteriole (diameter <50 μm) densities in ischaemic adductors of mice at 21 days post ischaemia (*n*=6 per group). For **b**–**e**, *P<0.05 versus *Ad.Null*; ^#^*P*<0.05 versus *Ad.p75*. Unpaired two-tailed Student’s *t*-test or Mann–Whitney nonparametric test was applied. All values are mean±s.e.m. of three independent experiments.

We previously demonstrated that diabetic *p75*^*NTR*^ knockout mice (p75KO), with surgically induced limb ischaemia, show improved post-ischaemic angiogenesis and blood flow recovery in comparison with the diabetic wild-type (WT) mice^19^. Conversely, local adenovirus-mediated overexpression of p75^NTR^ impairs post-ischaemic recovery in non-diabetic WT mice (compared with non-diabetic WT mice injected with Ad.*Null*)^11^. The miR-503 expression was increased in the ischaemic muscles of diabetic WT mice, but not in diabetic p75KO mice (Fig. 2a). Moreover, *Ad.p75* increased miR-503 expression in non-diabetic WT, with this response being blunted by simultaneous injection of *Ad.decoy503* (Fig. 2b). Importantly, *Ad.decoy503* reduced the negative impact of *Ad.p75* (versus *Ad.Null*) on post-ischaemic recovery by improving reperfusion (Fig. 2c), increasing the capillary and arteriole densities in the ischaemic muscles (Fig. 2d,e), and reducing the number of necrotic toes (Supplementary Fig. 4A). Finally, the analysis of expression of *CDC25A* and *CCNE1*, previously identified target genes of miR-503 (ref. 10), confirmed that *Ad.p75* injection alone inhibits their mRNA level; however, this effect was reverted by the co-injection of *Ad.p75* and *Ad.decoy503* (Supplementary Fig. 4B,C). These results confirmed target gene regulation by miR-503, as previously published in the diabetic mouse model of limb ischaemia^10^.

### NF-κB p65-dependent transactivation of miR-503

**Figure 3:**
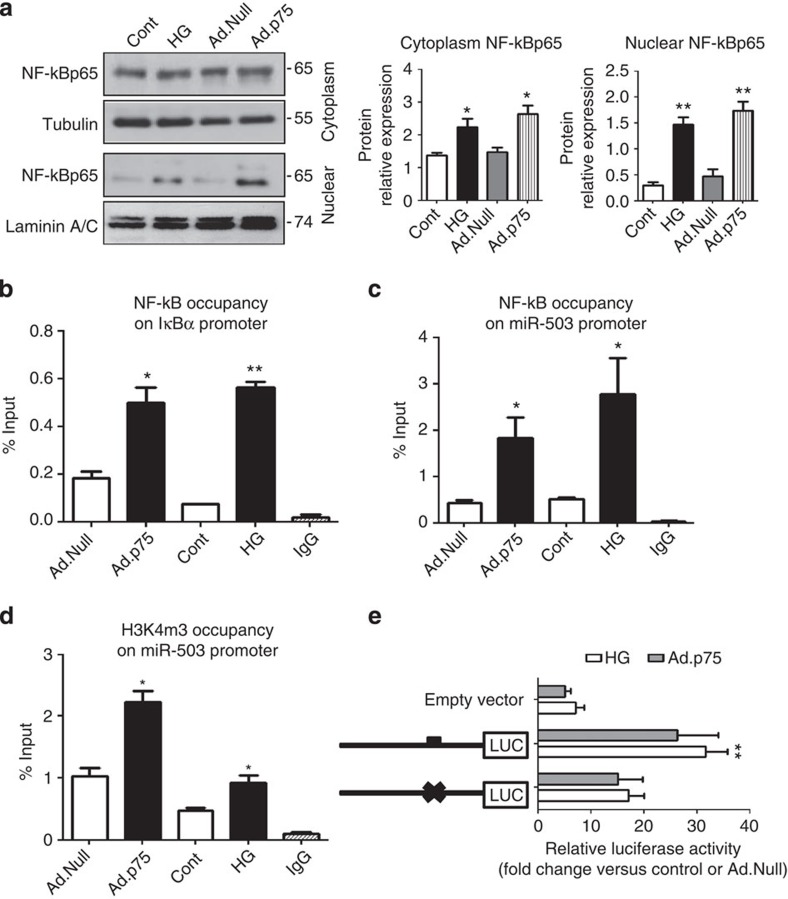
NF-κB p65 binds miR-503 promoter and regulates its transcription. (**a**) HUVECs were treated with HG (control: L-Glucose) or transduced with *Ad.p75* (control: *Ad.Null*). Representative western blot images and protein quantification of NF-κB p65, Tubulin and Laminin in nuclear and cytoplasmatic cell extracts *P<0.05, **P<0.01 versus Cont (*n*=3). (**b**) Sonicated chromatin of samples prepared as above was subjected to ChIP using normal mouse IgG or anti-NF-κB p65 antibody. Nuclear translocation of NF-κB p65 was verified with qPCR using primers for the IKBα promoter as a positive locus. (**c**) NF-κB p65 enrichment of miR-503 promoter (−3,480 bp from TSS). (**d**) Occupancy of H3K4me3 in the region of the NF-κB p65-binding sequence within the miR-503 promoter. For **b**–**d**, IgG-precipitated samples were used as negative control. Data are presented as % input for each IP sample relative to the input chromatin (1%) for each amplicon and ChIP sample as indicated; *P<0.05 versus Cont or *Ad.Null*; **P<0.01 versus Cont (*n*=3). (**e**) HUVECs were transfected with various luciferase reporter constructs spanning the putative NF-κB-binding sites of the miR-503 promoter and treated in the above conditions. Luciferase activity was measured and presented as a fold-change compared with osmotic control or *Ad.Null*. *P<0.05 versus empty vector (*n*=5). Unpaired two-tailed Student’s *t*-test or Mann–Whitney nonparametric test was applied. All values are mean±s.e.m. of three independent experiments.

Bioinformatics analysis of miRNAs upregulated by p75^NTR^ has revealed enrichment for miRNAs transcribed by NF-κB. In particular, miR-30c-2* (ref. 20), miR-198 (ref. 21) and miR-183* (ref. 22) were previously identified as NF-κB targets. In line with this, we tested whether NF-κB could also mediate transcription of miR-503 in response to increased p75^NTR^. We thus performed sequence analysis of the human miR-503 hypothetical promoter at chromosome X (5,000-bp region spanning the transcription starting site (TSS) in the genomic location ChrX:133,681,808 (ref. 23)), and identified a putative binding site for NF-κB p65 at −3,480 bp upstream of the TSS. Interestingly, both p75^NTR^ overexpression and HG induced the nuclear translocation of NF-κB p65 subunit in ECs (Fig. 3a). ChIP assay confirmed the binding of NF-κB p65 to the IKBα promoter (positive control for NF-κB translocation, Fig. 3b). Further ChIP assays were carried out to investigate whether NF-κB p65 binds to the promoter regions of miR-503. Under HG, or after *p75*^*NTR*^ overexpression (*Ad.p75*), we observed an increased binding of NF-κB p65 to the *miR-503* promoter compared with L-Glucose or *Ad.Null*, respectively (Fig. 3c). Trimethylation of histone H3 at lysine 4 (H3K4me3) positively correlates with transcriptional activation of gene expression^24^. Accordingly, we detected an enrichment of H3K4me3 at the TSS of the miR-503 promoter in ECs infected with *Ad.p75* or exposed to HG in comparison with respective controls (Fig. 3d). Lastly, p75^NTR^ overexpression or HG treatment in ECs induced a significant upregulation in luciferase activity of the reporter construct containing NF-κB-binding site within miR-503 promoter sequence. Mutation of a putative NF-κB-binding site prevented this upregulation of luciferase activity under the above conditions, thus showing that binding of NF-κB results in miR-503 transcription (Fig. 3e).

### dnIKK2 restores angiogenesis in diabetic ischaemic mice

**Figure 4:**
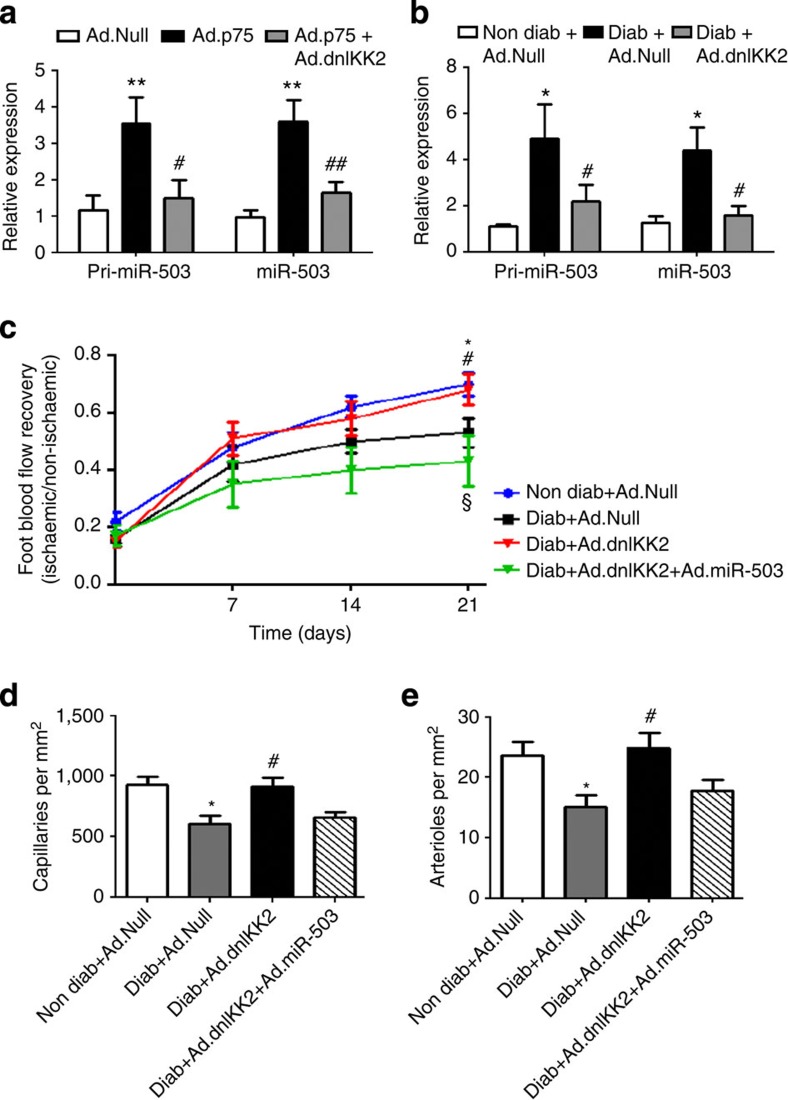
*In vivo* NF-κB-dependent transcription of miR-503. (**a**) HUVECs were transduced with *Ad.Null*, *Ad.p75* or *Ad.dnIKK2*, and qPCR was carried out to measure the expression of pri-miR-503 and mature miR-503; **P<0.01 versus *Ad.Null*; ^#^*P*<0.05, ^##^*P*<0.01 versus *Ad.p75* (*n*=3). (**b**) Relative expression of miR-503 in the ischaemic adductors of non-diabetic and diabetic mice, which received *Ad.Null*, *Ad.dnIKK2 or Ad.miR-503* (*n*=6 per group). Ad.*Null* was given to singly injected mice to equalize the virus quantity. (**c**) Line graph shows the time course of post-ischaemic foot blood flow recovery in mice (calculated as the ratio between ischaemic and contralateral foot blood flow; *n*=12 per group). (**d**,**e**) Column graphs show capillary and small arteriole (diameter <50 μm) densities in the ischaemic adductors of mice at 21 days post-ischaemia (*n*=8 per group). For **b**–**e**, *P<0.05 versus Non Diab*+Ad.Null*; ^#^*P*<0.05 versus Diab*+Ad.Null*; ^§^*P*<0.05 versus Diab+*Ad.dnIKK2*. Unpaired two-tailed Student’s *t*-test or Mann–Whitney nonparametric test was applied. All values are mean±s.e.m. of three independent experiments.

To further validate the role of NF-κB in regulating *miR-503* transcription in response to HG or p75^NTR^ overexpression, we used a loss-of-function approach. In particular, we asked whether a dominant-negative form of IkB kinase 2 (dnIKK2)^25^, a kinase that is an upstream activator of NF-κB, interferes with the expression of miR-503. In cultured ECs, *Ad.dnIKK2* reduced the pri-miR-503 and mature miR-503 expression, which were previously increased by *Ad.p75* (Fig. 4a) or HG (Supplementary Fig. 5A). In addition, local delivery of *Ad.dnIKK2* dramatically reduced miR-503 expression in the ischaemic limb muscles of diabetic mice (Fig. 4b). Moreover, *Ad.dnIKK2* rescued the blood flow recovery (Fig. 4c and Supplementary Fig. 5B), increasing the capillary and arteriole densities in ischaemic limb muscles of diabetic mice (Fig. 4d,e, respectively), and reduced the number of necrotic toes (Supplementary Fig. 5C). These positive effects of *Ad.dnIKK2* on vascularization were abolished by the simultaneous overexpression of miR-503 (by adenovirus carrying miR-503; *Ad.miR-503*; Fig. 4c–e and Supplementary Fig. 5B,C). Taken together, the above evidence suggests that regulation of NF-κB has a strong impact on post-ischaemic vascularization in diabetic mice via the regulation of miR-503 expression.

### Mechanism of MPs-miR-503 release from ECs

**Figure 5:**
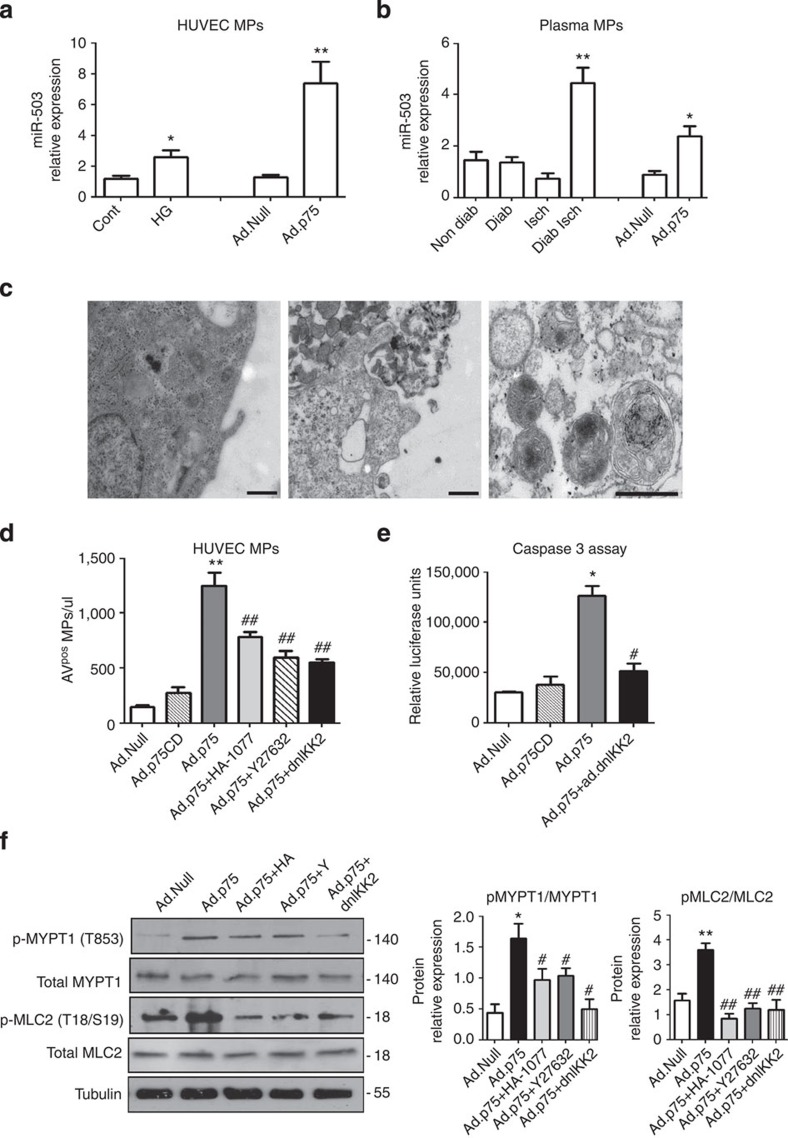
Mechanism of MP-miR-503 release in endothelial cells. Expression of mature miR-503 in MPs isolated by centrifugation from (**a**) the supernatant of HUVECs transduced with *Ad.Null* or *Ad.p75*; or exposed to HG versus L-Glucose control (**P<0.01, *P<0.05 versus Cont or *Ad.Null; n*=3). (**b**) From the plasma of non-diabetic, diabetic, non-diabetic ischaemic and diabetic ischaemic mice or ischaemic mice after *Ad.Null* or *Ad.p75* overexpression (**P<0.01, *P<0.05 versus Non-Diab; *P<0.05 versus *Ad.Null*, *n*=8 per group). (**c**) Transmission electron microscopy showing the shedding of MPs by a membrane-blebbing process in *Ad.Null-* (left) and *Ad.p75* (middle)-transduced HUVECs; (right) purified endothelial MPs showing a spheroid shape. Scale bars, 500 nm. (**d**) Flow cytometric analysis of MPs from HUVECs transduced with the *Ad.Null*, *Ad.p75, Ad.dnIKK2* or treated with Rho kinase inhibitors Y27632 (10 μM) and HA-1077 (10 μM). (**e**) Caspase-3 activity in HUVECs transduced with *Ad.Null*, *Ad.p75* or *Ad.dnIKK2*. (**f**) Representative western blot analyses and protein quantification of phospho and total MLC2 and MYPT1 in HUVECs transduced with *Ad.Null*, *Ad.p75*, *Ad.dnIKK2* or treated with Rho kinase inhibitors Y27632 and HA-1077. For **d**–**f**, *P<0.05, **P<0.01 versus *Ad.Null*, ^#^*P*<0.05, ^##^*P*<0.01 versus *Ad.p75* (*n*=3). Unpaired two-tailed Student’s *t*-test or Mann–Whitney nonparametric test was applied. All values are mean±s.e.m. of three independent experiments.

Following p75^NTR^ overexpression, the release of MPs carrying miR-503 from the cultured ECs increased (Fig. 5a). To this end, we also found that condition that mimics diabetes and ischaemia *in vitro* (HG in low-growth-factor medium) increased the expression of miR-503 within MPs (Fig. 5a). In addition, miR-503 was present in the MPs from the plasma of diabetic ischaemic mice and non-diabetic ischaemic mice following adenovirus-mediated overexpression of p75^NTR^ (Fig. 5b). Transmission electron microscopy on HUVECs transduced with *Ad.Null* or *Ad.p75* confirmed the shedding of MPs by a membrane-blebbing process, which is typical of MPs (Fig. 5c). The MPs collected from the medium showed a heterogeneous pattern of particles, with ∼70% of MPs being 200–500 nm in size (Supplementary Fig. 6A). Then, we used flow cytometry to analyse and quantify MPs, and we showed that p75^NTR^ increased the release of a well-defined population of HUVEC-derived MPs, which were expressing Annexin-V (AnnV^Pos^) on their extracellular surface (Supplementary Fig. 6B). Further analysis of expression of vascular miRNAs showed that miR-126, miR-143 and miR-145 are not upregulated in the ECs nor released into the endothelial MPs following overexpression of p75^NTR^ (Supplementary Fig. 6C,D).

Next, we examined the possible mechanism of p75^NTR^-related release of endothelial MPs. Previous studies have identified the role of Rho kinases (ROCK)^26^,^27^ and NF-κB pathways^28^ in the biogenesis of MPs and their release in different cellular contexts. Following p75^NTR^ overexpression, either the inhibitors of ROCK (Y27632 or HA-1077) or NF-κB (*dnIKK2*) decreased the release of MPs (Fig. 5d). The apoptotic membrane blebbing, a core event in the shedding of MPs, can also be induced by caspase-3-mediated activation of ROCK and subsequent myosin light-chain (MLC) phosphorylation^29^. Since p75^NTR^ overexpression induces caspase-3-dependent apoptosis in ECs^11^, we analysed the regulation of caspase-3 activity by NF-κB. Transduction of HUVECs with *Ad.dnIKK2* to inhibit NF-κB blocked p75^NTR^-mediated caspase-3 activation (Fig. 5e). Overexpression of a truncated form of p75^NTR^ (p75^NTR^ cytoplasmatic domain, p75CD, missing the death domain and thus not able to activate caspase-3 (ref. 30)) failed to promote the release of endothelial MPs (Fig. 5d,e). In addition, inhibition of NF-κB decreased p75^NTR^-mediated phosphorylation of MLC2 and myosin phosphatase target protein (MYPT1), another ROCK substrate^31^, with an effect comparable to treatment with ROCK inhibitors, Y27632 and HA-1077 (Fig. 5f). Taken together, these results demonstrate that NF-κB activation is a major determinant in caspase-3/ROCK-mediated release of MPs on activation of p75^NTR^. Similarly to *Ad.p75*, the exposure to HG has induced shedding of MPs in HUVECs, and this response was inhibited by Y27632, HA-1077 or *Ad.dnIKK2* (Supplementary Fig. 6E,F). Moreover, EC apoptosis was reduced by *Ad.dnIKK2* (Supplementary Fig. 6G).

### miR-503 regulates EFNB2 and VEGFA expression in pericytes

**Figure 6:**
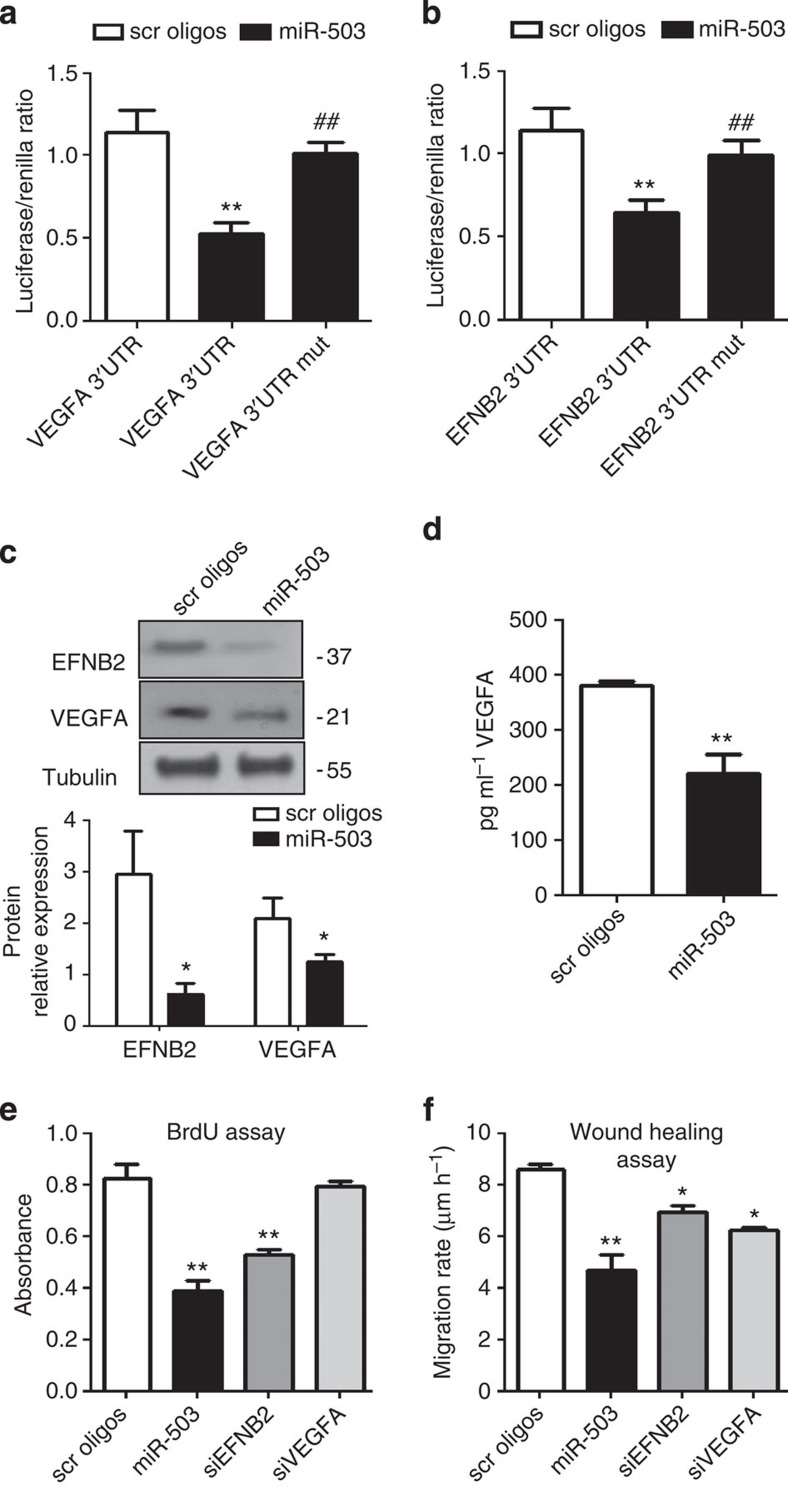
*EFNB2* and *VEGFA* are miR-503 target genes. (**a**,**b**) Luciferase activity at 48 h post-co-transfection of HEK293T cells with either miR-503 or miR-scrambled oligonucleotides (scr oligos) and the following plasmids: 3′-UTR-EFNB2, 3′-UTR-VEGFA, 3′-UTR-EFNB2mut and 3′-UTR-VEGFAmut (with mutation of the putative miRNA target site). **P<0.01 versus scr oligos; ^##^*P*<0.01 versus non-mutated (*n*=5). (**c**) Representative western blot images and protein quantification of VEGFA and EFNB2). Tubulin was used as a loading control. (**d**) VEGFA quantification in the medium by ELISA. (**e**) Proliferation (assessed by 5-bromodeoxyuridine incorporation) or (**f**) migration of pericytes transfected with miR-503 or siRNA oligos for *EFNB2* or *VEGFA* (control: scr oligos). For **c**–**f**, *P<0.05; **P<0.01 versus scr oligos (*n*=3). Unpaired two-tailed Student’s *t*-test or Mann–Whitney nonparametric test was applied. All values are mean±s.e.m. of three independent experiments.

The Targetscan 6.2 algorithm^32^ predicted *EFNB2* and *VEGFA* to be the direct targets of miR-503. Accordingly, both targets were predicted to contain a single conserved binding sequence for miR-503 in their 3′UTRs (Supplementary Fig. 7A). *EFNB2* controls interactions between the pericytes and endothelium, and their motility^33^. Moreover, pericytes promote survival of ECs through paracrine VEGFA signalling^34^. Then, to investigate whether miR-503 directly binds the 3′UTRs of *EFNB2* and *VEGFA*, we performed a luciferase reporter assay in which luciferase, the reporter gene, was fused to the WT 3′UTRs of *EFNB2* or *VEGFA*, respectively. Overexpression of miR-503 (Supplementary Fig. 7B) decreased luciferase activity for each of the putative target genes, whereas mutation of the putative miR-503-binding site within *EFNB2* or *VEGFA* prevented miR-503-induced reduction in luciferase activity (Fig. 6a,b). Moreover, miR-503 overexpression reduced the target gene mRNA (Supplementary Fig. 7C) and protein levels (Fig. 6c), and the secretion of VEGFA in cell medium (Fig. 6d). Next, we investigated the role of miR-503 on the functional properties of pericytes. Overexpression of miR-503 reduced the proliferative and migratory capacities of pericytes (Fig. 6e,f). Following silencing of *EFNB2* in pericytes, we observed reduced proliferation and migration, whereas silencing of *VEGFA* reduced only the migration of pericytes (Fig. 6e,f). Finally, we investigated whether EFNB2 and VEGFA are required for miR-503-induced regulation of migration and proliferation in pericytes. To this end, miR-503 was overexpressed in the presence of *EFNB2 or VEGFA* cDNA, which lacked parts of the 3′UTR sequence (*ΔEFNB2* and *ΔVEGFA*) containing the binding sites for the miR-503 (and therefore could not be targeted by miR-503). The results showed that overexpression of *ΔEFNB2* (Supplementary Fig. 8A,C) or *ΔVEGFA* (Supplementary Fig. 8B,D) has partially restored migration and proliferation capacities in pericytes.

### MPs transport miR-503 from ECs into pericytes

**Figure 7:**
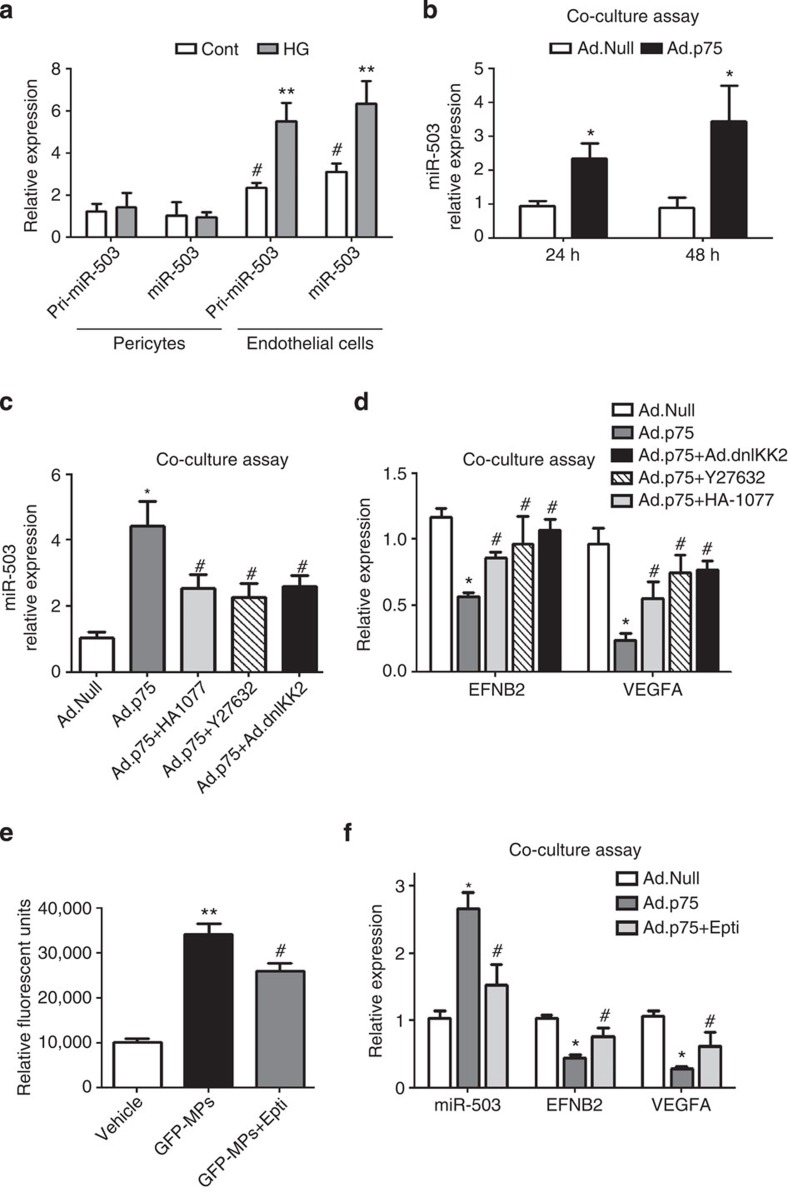
miR-503 targets *VEGFA* and *EFNB2* in pericytes. (**a**) Expression of pri-miR-503and mature miR-503 was measured in ECs and pericytes cultured in HG; **P<0.01 versus control endothelial cells; ^#^*P*<0.05 versus control pericytes (*n*=3). (**b**) *In vitro* coculture system of HUVECs overexpressing *p75*^*NTR*^ (or *Ad.Null* as control; top compartment) with pericytes (bottom compartment) has been set up, in which the cells are separated by a membrane to prevent direct cell–cell contact. miR-503 relative expression in pericytes was analysed using qPCR at 24 and 48 h after the start of coculture; *P<0.05 versus *Ad.Null* (*n*=3). (**c**) *Ad.Null-* or *Ad.p75*-transduced HUVECs (top compartments) were co-transduced with *Ad.dnIKK2* or treated with Y27632 or HA-1077 and miR-503 expression was analysed in pericytes (bottom compartment) after 48 h from the treatment; (**d**) in the same experimental conditions, expression of *EFNB2* and *VEGFA* was measured; *P<0.05 versus *Ad.Null*; ^#^*P*<0.05 versus *Ad.p75* (*n*=3). (**e**) The measurement by fluorimeter of the uptake of endothelial GFP-labelled MPs by pericytes. Where stated, pericytes were incubated with Eptifibatide (250 μM). **P<0.01 versus vehicle; ^#^*P*<0.05 versus GFP-MPs (*n*=5). (**f**) Expression levels of miR-503, *EFNB2* and *VEGFA* were measured in the coculture system described in **b**,**c** in the presence of Eptifibatide. *P<0.05 versus *Ad.Null*; ^#^*P*<0.05 versus *Ad.p75* (*n*=3). Unpaired two-tailed Student’s *t*-test or Mann–Whitney nonparametric test was applied. All values are mean±s.e.m. of three independent experiments.

Then, we investigated whether endothelial MPs containing miR-503 could transfer miR-503 from ECs into pericytes and thus reduce the expression of miR-503 target genes in the recipient cells. Under basal conditions, pericytes express miR-503, although in a lower amount compared with ECs. However, treatment with HG in low-growth-factor medium did not increase miR-503 transcription (pri-miR-503 levels) or the levels of mature miR-503 in pericytes (Fig. 7a). Direct incubation of pericytes with the endothelial MPs carrying miR-503 increased the intracellular levels of miR-503 in a concentration-dependent manner (Supplementary Fig. 9A). To then determine whether endothelial MPs could be transferred into pericytes in a paracrine manner, we employed a coculture system with HUVECs on top and pericytes at the bottom, separated by a membrane to prevent direct cell–cell contact. As noted before, p75^NTR^ overexpression induced the release of MPs carrying miR-503 in HUVECs (Fig. 5a). HUVECs were labelled with 3,3′-dioctadecyloxacarbocyanine (green fluorescence) and transduced with *Ad.p75* or *Ad.Null*. In this coculture system, green-labelled endothelial MPs were transferred from the p75^NTR^-HUVECs into pericytes (Supplementary Fig. 9B) and increased expression of miR-503 was detected in the pericytes (Fig. 7b). Consistently, the inhibition of ROCK (by Y27632 or HA-1077) or NF-κB (*dnIKK2*), previously shown to decrease the release of MPs (Fig. 5d), has reduced the transfer of miR-503 into pericytes exposed to the MPs (Fig. 7c), also suggesting that this process is mediated by actively formed endothelial MPs. Moreover, transfer of endothelial MPs carrying miR-503 into pericytes reduced the expression of *EFNB2* and *VEGFA*, whereas use of ROCK or NF-κB inhibitors has prevented the downregulation of the two miR-503 target genes (Fig. 7d).

Several studies demonstrated that incorporation of MPs in target cells required the engagement with integrins^35^,^36^. Taking this into consideration, we have analysed the effect of Eptifibatide, an antagonist of β3 integrins^37^, on the uptake of GFP-labelled MPs by pericytes (Fig. 7e). The uptake of MPs was seen through the increase of GFP protein in the pericytes and was partially reduced because of treatment with Eptifibatide (250 μm; Fig. 7e). Furthermore, using a coculture system described above, we have demonstrated that treatment with Eptifibatide prevented the transfer of miR-503 through MPs from ECs into pericytes (Fig. 7f), preventing the downregulation of target genes, *VEGFA* and EFNB2 by miR-503 (Fig. 7f). In conclusion, the crosstalk between ECs and pericyte MPs could be in part regulated by the integrin signalling.

### The *in vivo* transfer of miR-503 between ECs and pericytes

**Figure 8:**
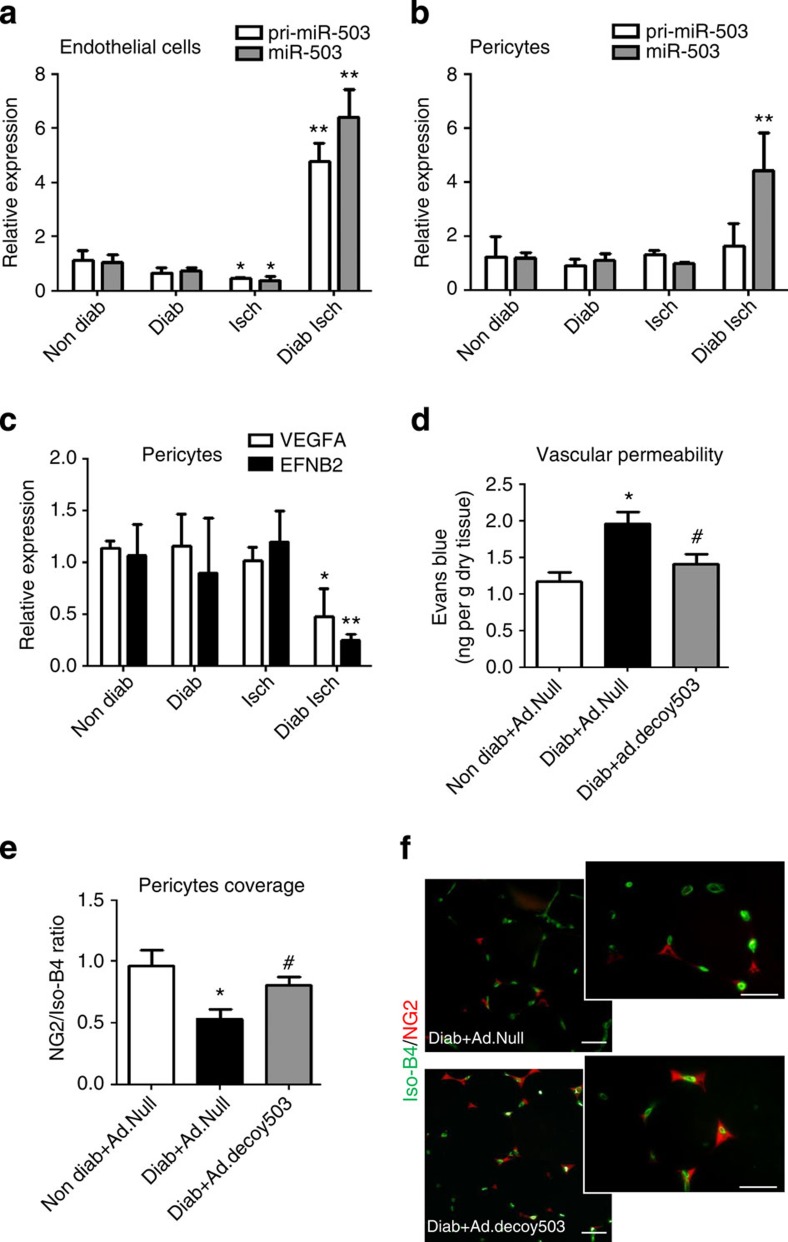
*In vivo* transfer of miR-503 during diabetes and ischaemia. Unilateral limb ischaemia was induced in diabetic mice and endothelial cells, and pericytes were sorted from the limb muscles using CD31 and NG2 antibodies. Relative expression of pri-miR-503 and mature miR-503 was measured in (**a**) endothelial cells and in (**b**) pericytes. (**c**) Relative expression of *EFNB2* and *VEGFA* in pericytes sorted from the limb muscles. For **a**–**c**, *P<0.05, **P<0.01 versus Non Diab (*n*=10 per group). (**d**) Quantitative analysis of vascular permeability using Evans blue dye and expressed as ng of dye per mg of muscle tissue (*n*=8 per group). *P<0.05 versus Non Diab+*Ad.Null*; ^#^*P*<0.05 versus Diab*+Ad.Null.* (**e**) Representative Isolectin-B4 staining (green) and immunostaining with anti-NG2 antibody (red) in adductor muscle of diabetic ischaemic and diabetic ischaemic mice injected with *Ad.decoy503*. Scale bar, 100 μm. (**f**) Quantification of pericyte coverage determined as ratio of NG2 to Isolectin-B4 (Iso-B4) staining. *P<0.05 versus Non Diab+*Ad.Null*; ^#^*P*<0.05 versus Diab+*Ad.Null* (*n*=6 per group). Unpaired two-tailed Student’s *t*-test or Mann–Whitney nonparametric test was applied. All values are mean±s.e.m. of three independent experiments.

On the basis of our *in vitro* findings we additionally explored whether transfer of miR-503 from ECs into pericytes could repress *EFNB2* and *VEGFA in vivo*. To this aim, ECs and pericytes were sorted using CD31 and NG2 antibodies, respectively, from the ischaemic and non-ischaemic limb muscles of diabetic and non-diabetic mice (Supplementary Fig. 10. In ECs extracted from ischaemic limbs of diabetic mice, the pri-miR-503 and mature miR-503 are more expressed in comparison with non-diabetic muscle (Fig. 8a), whereas diabetes and ischaemia did not increase the transcription of pri-miR-503 in the pericytes (Fig. 8b). Nonetheless, significant increase of expression of the mature miR-503 is observed in the pericytes under diabetic ischaemia (Fig. 8b), thus suggesting that mature miR-503 was likely to be acquired in a paracrine manner and not produced by the pericytes. In line with this, mRNA levels of *EFNB2* and *VEGFA* were significantly lower in the sorted pericytes from diabetic muscles following limb ischaemia in comparison with non-diabetic muscles (Fig. 8c). Reduced *EFNB2* expression has been associated with the loss of pericytes, leading to excessive vascular permeability^33^. Therefore, we evaluated the impact of local miR-503 inhibition (by *Ad.decoy503*) on vascular permeability in the ischaemic muscles of diabetic mice, using *Ad-Null*-infected ischaemic muscles from non-diabetic mice as additional reference (Fig. 8d).

Within diabetic ischaemic muscles, there was a substantial increase in the retention of Evans blue dye, demonstrating an increased vascular permeability. This could be prevented by local inhibition of miR-503 (Fig. 8d). Because pericyte coverage often correlates with vascular permeability^38^, we have quantified the coverage of pericytes in capillaries by NG2 and isolectin-B4 co-staining. In agreement with the observed increased permeability, there was a significant decrease in the pericyte coverage of the neovasculature in diabetic ischaemic limb muscles, whereas *decoy503* overexpression has restored pericyte density close to the ischaemic control (Fig. 8e,f).

Finally, we employed *in situ* hybridization to further confirm the localization of miR-503 in ECs and pericytes in the limb muscles of diabetic ischaemic mice (Supplementary Fig. 11). A triple staining was performed using a miR-503 probe, isolectin-B4 and NG2. Absence of the staining using the scramble sequence probe shows the success of the protocol with this technique (Supplementary Fig. 11A). MiR-503 was not detectable in the non-diabetic controls (Supplementary Fig. 11B), but localized to ECs, pericytes and myocytes within diabetic ischaemic muscles (Supplementary Fig. 11C).

**Figure 9:**
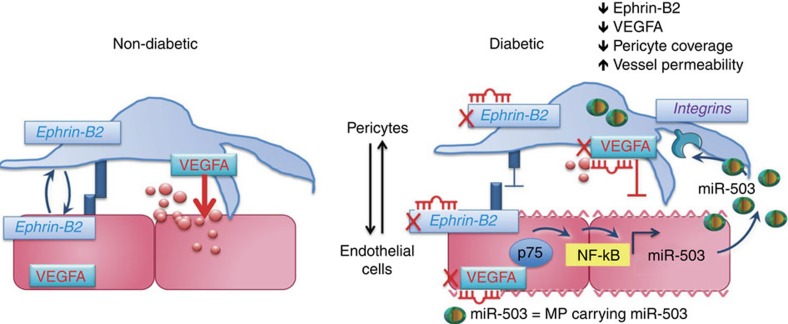
Proposed mechanism of crosstalk between ECs and pericytes during microvascular diabetic complications. p75^NTR^-dependent activation of NF-κB regulates miR-503 transcription in hyperglycemic ECs and the release of miR-503 in the extracellular compartment within microparticles. Microparticles carrying miR-503 are secreted from the diabetic ECs and can be transferred into neighbouring pericytes to subsequently modulate vessel permeability and angiogenesis through miR-503 target genes, *VEGFA* and *EFNB2*.

Figure 9 presented the proposed mechanism behind the crosstalk between ECs and pericytes during microvascular complications of diabetes.

## Discussion

Our study provides the first insights into the mechanisms of transcriptional regulation of *miR-503* in ECs by diabetes. We additionally show the first evidence of the release and trafficking of endothelial MPs from ECs into pericytes, thus MP mediated the transfer of miR-503 during the diabetes-induced vascular disease. Previously, we defined neurotrophin receptor p75^NTR^ and miR-503 as independent negative modulators of EC function and diabetes-induced post-ischaemic reparative neovascularization^10^,^11^. Here we have further explored and established the mechanisms that regulate miR-503 expression through p75^NTR^ activation of NF-κB; associated them with the negative effects diabetes induces on vascular cells. Moreover, we have demonstrated that miR-503 can be transferred via endothelial MPs to produce a negative effect in the neighbouring pericytes. To our knowledge, this is the very first example of a direct involvement of miR-503 signalling in the endothelial–pericyte crosstalk under diabetic ischaemia.

Given that the cytoplasmatic domain of p75^NTR^ does not contain a catalytic motif, the signal mediation depends on the interaction with cytoplasmic proteins; complex signalling, however, makes it difficult to formulate a unified functional model downstream of this receptor^13^. We provide mechanistic evidence that NF-κB plays a primary role in regulating miR-503 transcription in ECs, exposed to HG or overexpressing p75^NTR^. Moreover, the activation of NF-κB determines the release of miR-503 within the endothelial MPs, which can be found in the extracellular compartment *in vitro* and in the systemic circulation of diabetic ischaemic mice. P75^NTR^ mediates nuclear translocation of the NF-κB p65 subunit to bind the promoter of miR-503 and induce transcription of miR-503. Moreover, NF-κB-dependent p75^NTR^ regulation of apoptosis further induces ROCK activation, leading to blebbing of cellular membrane and shedding of endothelial MPs. NF-κB activation is sufficient to control the transcriptional regulation of miR-503 and its release into MPs. Interestingly, recent *in vitro* studies have clarified the involvement of epigenetic mechanisms in the modulation of glucose-induced gene expression of the key subunit of NF-κB p65, with subsequent effects on the NF-κB activation^39^. This opens up the possibilities for novel integrative approaches to understand the pathogenesis of diabetic vascular complications.

There is currently a large interest in signalling pathways operating during EC–pericyte crosstalk^18^,^40^; therefore, future investigation will provide crucial information on the paracrine molecular mechanisms controlling capillary formation.

We have initially identified VEGFA and EFNB2 as target genes of miR-503, and both of these targets are critical for EC–pericyte crosstalk. Pericytes produce VEGFA, which promotes EC survival and guides migration and angiogenesis^34^,^41^. Ephrin-Eph receptor signalling plays major roles in ECs but has also been implicated in mural cell biology. A mural cell-specific knockout of *efnb2* resulted in poor association of vascular smooth muscle cells and pericytes within vessels of different sizes^33^. Moreover, *efnb2* is a critical mediator of endothelial-to-pericyte assembly during the postnatal vascular remodelling^42^.

Despite the tight association between the ECs and pericytes in microcirculation, and their interdependent nature, the mechanisms of EC–pericyte interaction in diabetes are largely unknown. However, a striking correlation exists between the pericyte coverage and microvessel stabilization^43^ as well as the microvascular permeability. Several studies demonstrated that pericyte deficiency causes increased vessel permeability (for example, in PDGF-B/PDGFR-b mutant mice), the degree of which correlated directly with density of pericytes^44^,^45^. Hence, pericytes regulate EC-to-cell junctions and EC behaviour^46^, which confers these mural cells with a greater biological relevance than being just supportive cells.

In addition, the decrease of pericyte coverage in the microcirculation precedes capillary loss as already described in diabetic retinopathy^47^ and in the pancreatic islet^48^. In line with this, we can hypothesize that loss of ECs during diabetic ischaemia in limb muscles could be in part a consequence of the lost EC–pericyte interaction.

Here we demonstrated that decrease in pericyte coverage correlated with increased permeability in limb muscles of diabetic ischaemic mice. Overexpression of *decoy503* in the ischaemic limb muscles of diabetic mice has restored pericyte coverage close to the ischaemic control. Overall, we propose that miR-503 upregulation within the ECs affects the pericyte functionality and *in vivo* coverage, which correlates with the increased vascular permeability in a diabetic ischaemic mouse model.

Beside soluble factors, receptor-mediated events and direct cell-to-cell contact, more recent studies have suggested that cells may also communicate by EVs^49^. Two distinct processes of EV release from the cells have been described so far: (1) they may derive from the endosomal membranes that are extruded from the cell surface of activated cells as exosomes^50^ and, alternatively, (2) EVs may take origin by direct shedding from the cell plasma membrane as MPs. The MPs released from ECs are named endothelial MPs and are identified by carrying EC protein markers and by binding to the apoptosis marker Annexin-V (ref. 17). Endothelial MPs are regarded as a possible biomarker of EC dysfunction, and their circulating numbers are elevated in diabetic patients with endothelial dysfunction^51^. We have previously demonstrated that in ECs p75^NTR^ promotes the release of endothelial MPs^11^. Now, we provide new evidence that MPs carrying miR-503 are secreted from diabetic ECs and can transfer miR-503 into the neighbouring pericytes, thus modulating gene expression and their biological phenotype. Our data show that transcription of pri-miR-503 is not increased in the pericytes under ischaemic condition in diabetic mice. Moreover, in our *in vivo* experiment, we detected only the mature form of miR-503 in the pericytes, which might be reconciled with a direct transfer of mature miR-503 from the endothelial MPs into pericytes. Then, our study extends these findings by establishing that endothelial MPs carrying miR-503 interfere with *EFNB2* and *VEGFA* expression in pericytes, further blocking post-ischaemic angiogenesis and vascular integrity under diabetes.

The discovery of an endothelial MP-mediated delivery of miR-503 into pericytes raises intriguing possibilities to better understand the mechanisms behind various models of vascular cell-to-cell communications.

One of the most important and unknown aspects of MP function concerns how MPs, on being shed from ECs, target recipient cells and transfer their cargo. Potential mechanisms previously described have shown that MPs target their recipient cells through interaction with integrins^35^ and phosphatidylserine receptors^52^. Currently, it is unclear whether specific receptors on ECs or pericytes take up MP–miRNA complex and which mechanisms are at play to load functional miRNAs into MPs.

In this study, we have demonstrated that the uptake of endothelial MPs into pericytes is partially mediated by β3 integrins. Integrins are expressed not only in ECs but also on the surface of pericytes^53^. Being that endothelial MPs express the adhesion molecules of their parent cells^54^, the inhibition of their uptake by pericytes using Eptifibatide can be attributed to the blockade of β3 integrins either on MP and/or pericyte surface. Future studies are required to demonstrate the involvement and to delineate the specific mechanisms behind MP–miRNA communication *in vivo*.

In conclusion, our data demonstrate a novel mechanism in diabetic ischaemia, involving coordinated expression of p75^NTR^ and regulation of miR-503 in ECs, and thus leading to impaired function of pericytes following uptake of the endothelial MPs carrying miR-503.

## Methods

### Cells and cell culture

HUVECs and HMVECs (both from Lonza) were grown in EGM-2 (EBM-2 medium supplemented with growth factors and normal 5 mM D-Glucose, NG) and 2% fetal bovine serum (FBS; Lonza). To mimic diabetes and ischaemia *in vitro*, the ECs were maintained in EBM-2 (growth factor-free medium) with 2% FBS and 25 mM D-glucose (HG). L-Glucose was used as an osmotic Cont at the concentration of 25 mM. HUVECs were used between P2 and P6 passages. HA-1077 or Y27632 (Sigma) have been used at concentration of 10 μM; Eptifibatide (Sigma) has been used at concentration of 250 μM. HEK293T cells (ATCC, CRL-11268) were cultured in D-MEM with 10% FBS (Life Technologies). Pericytes were grown on fibronectin (10 μg ml^−1^)-coated plates and maintained in EGM-2 with 2% FBS.

### Pericyte isolation

Pericyte progenitor cells were isolated from vein leftovers of patients undergoing coronary artery bypass graft surgery or varicose vein removal, as previously described^55^. In brief, saphenous veins were carefully dissected from surrounding tissues using a sterile scalpel and then thoroughly washed in PBS. Veins were manually minced before 4-h incubation with 3.7 mg ml^−1^ Liberase 2 (Roche) and filtered passing the cell suspension through a 30-μm cell strainer. Cells were depleted for ECs with anti-CD31-conjugated beads (Miltenyi), according to the manufacturer’s instruction. The remaining cells were purified by selecting CD34+ cells by anti-CD34 beads (Miltenyi). Target cells were then plated on fibronectin (10 μg ml^−1^)-coated plates in presence of differentiation medium (EGM-2—2% FBS, Lonza). Adherent colonies were passaged to new culture dishes once they reached 60–70% confluence and frozen stocks generated after Passage 2 (P2) for the experiments shown in this publication. Trypsin-EDTA (Life Technologies) was utilized to detach cells from the growth substrate.

### Plasmid cloning

To clone EFNB2 or VEGFA, cDNAs, which lacked parts of the 3′UTR sequence and could not be targeted by miR-503 (EFNA2Δ or VEGFAΔ), EFNA2 (NCBI accession: NM_004093) and VEGFA cDNA (NCBI accession: NM_001025366), were excised from pCMV6-XL4 (Origene) using NotI/XbaI or NotI/SmaI, respectively and cloned in pcDNA3.1 using standard techniques. Thus, the last 2,834 bps of EFNA2 3′UTR or 1,705 bps of VEGFA 3′UTR, encompassing miR-503-binding sites, were deleted. Plasmids were transfected using Lipofectamine 3,000 (Invitrogen) according to the manufacturer’s instructions.

### Cell transfection, recombinant adenovirus and cell functional assay

Lipofectamine RNAiMAX (Invitrogen) was used to transfect HUVECs, HEK293T or pericytes with pre-miR-503, pre-miR-control (50 nM final concentration) or with short interfering RNA targeting p75^NTR^, EFNB2 or VEGFA, according to the manufacturer’s instructions. Adenoviral particles of human *p75*^NTR^ (ref. 11), Null, decoy503 (ref. 56), dnIKK2 (ref. 25) and miR-503 are produced and used as described in ref. 56. The following functional assays were performed: 5-bromodeoxyuridine incorporation assay using Cell Proliferation colorimetric assay (Roche); Caspase-activity assay using CaspaseGlo assay (Promega); migration assay and endothelial barrier function were performed using ECIS machine as described below. Matrigel assay with HUVECs was performed as previously described using BD Matrigel Basement Membrane Matrix (BD Biosciences)^56^.

### RNA extraction and quantitative real-time analysis

Total RNA was extracted using the miReasy kit (Qiagen). Real-time quantification to measure miRNAs was performed with the TaqMan miRNA reverse transcription kit and miRNA assay (miR-503 cat #001048, miR-183* cat#002270, miR-30c-2* cat#002110, miR-198 cat#002273, miR-30b* cat#002129, miR-658 cat#001513, miR-665 cat#002681, miR-371-5p cat#000559 and pri-miR-503 cat#Hs 03304160_pri; Applied Biosystems) using Lightcycler 480 (Roche). miRNA expression was normalized to the U6 small nucleolar RNA. For mRNA analysis, cDNA was amplified by quantitative real-time PCR (qPCR) and normalized to 18S ribosomal RNA. Each reaction was performed in triplicate. Quantification was performed by the 2^−ΔΔ*C*t^ method^57^. qPCR was used to measure p75^NTR^, EFNB2, VEGFA and 18S rRNA. Primers are predesigned from Sigma (KiCqStart Primers).

### Characterization and analysis of MPs

MPs were purified from cell culture media from ECs and from platelet-free plasma as previously described^11^. Briefly, confluent monolayers of HUVECs were grown and the supernatant was collected and pre-cleared by centrifugation at 1,500*g* for 15 min at 4 °C to remove cell debris. To pellet the extracellular particles (size, <1 μm), the supernatant was centrifuged at 20,500*g* for 1 h at 4 °C. The supernatant was removed and discarded, and the pelleted particles were washed with ice-cold PBS and repelleted by centrifugation at 20,500*g* for 1 h at 4 °C. Finally, the supernatant was removed and discarded, and the pelleted vesicles were resuspended in 700 μl Qiazol (Qiagen) for RNA isolation using the miRNeasy kit (Qiagen) following the instructions provided by the manufacturer or in EBM-2 for other experiments. For flow cytometry analysis, isolated extracellular particles were stained with an Annexin-V or p75^NTR^ antibody (BD Biosciences) and analysed by FACS^58^ with the FACSDiva software (BD Biosciences) on a FACS Canto II (BD Biosciences).

### miRNA array

Total RNA was extracted by using TRIzol (Invitrogen) according to the manufacturer’s instructions. Total RNAs (2.5 μg) were labelled with either Hy3 or Hy5, following the manufacturer’s protocol (miRCURY LNA microRNA Array Power labelling kit, Exiqon). Two-colour hybridization was carried out at 53 °C for 16 h using miRCURY LNA microRNA Arrays (miRBase v.10.0, Exiqon). Hybridization and washing steps were performed on the HS 400 PRO hybridization station (Tecan). The signal intensities were acquired by scanning the arrays on the GenePix 4,100-A microarray scanner (Molecular Devices, GenePix Pro 6.0.1.25 software). The obtained data were analysed by using the Limma package (Smyth, G.K. 2005) from the Bioconductor Project (Bioconductor 2.5 on R 2.10), performing background correction (normexp, cutoff=10) and within-array normalization (global LOESS) as well as between-array normalization (scale method). Differential expression and statistical significance were assessed by applying linear model fit and empirical Bayes method (lmFit, eBayes). The default table of Limma is ranked by B-statistics (B), closely related to the adjusted *P* value. The B-statistic used for this analysis is the log-odds that gene is differentially expressed and it is automatically adjusted for multiple testing by assuming that 1% of the genes are expected to be differentially expressed.

### ChIP

ChIPs were performed with confluent 15-cm tissue culture plates of HUVECs (Lonza, 2 × 10^6^ cells per condition). Cells were crosslinked for 10 min with formaldehyde (final concentration=1%). Glycine was added to a final concentration of 0.125 M and incubated for 5 min. Cells were washed 2 × with PBS and subsequently lysed in Chip Lysis Buffer (10 ml, 100 mM NaCl, 0.5% SDS, 5 mM EDTA, 50 mM Tris-HCl pH 8.1, 0.2% NaN_3_) supplemented with protease inhibitors and complete EDTA-free tablet. Cells were pelleted (2,100 r.p.m., 4 °C, 6 min) and resupsended in 300 μl of IP-dillution buffer (two parts ChIP lysis buffer and one part Triton dilution buffer: 100 mM NaCl, 5% Triton X-100, 5 mM EDTA, 100 mM Tris-HCl pH 8.6, 0.2% NaN_3_). Chromatin was sheared (3 × 60′′ON/30′′OFF) by sonication (Bioruptor UCD-200 ultrasound sonicator, Diagenode), resulting in DNA fragments between 200 and 500 bp in size. After centrifugation (14,000*g*, 15 min, 4 °C), between 20 and 50 μl of each sample was separated for de-crosslinking, diluted to 100 μl in ChIP elution buffer (1% SDS, 100 mM NaHCO_3_ and proteinase K) and left at 65 °C overnight to reverse crosslink for checking the DNA shearing. If sheared DNA was of the right size, 1% of chromatin sample was kept as Input. Chromatin was diluted to 200 μg ml^−1^ (for transcription factors) or 50 μg ml^−1^ (for histone modifications) concentration in the IP-dilution buffer. Immunoprecipitation was performed in 1 ml volume and 3–5 μg of test or control IgG antibody was added to the diluted chromatin and incubated overnight at 4 °C. The following day, immunocomplexes were collected with purified protein A-magnetic beads (Invitrogen) for 3 h at 4 °C with rotation. Beads were extensively washed 2 × in low salt (150 mM NaCl, 1% Triton X-100, 0.1% SDS, 2 mM EDTA pH 8.0, 20 mM Tris-HCl pH 8.0) followed by 1 × in high-salt buffer (500 mM NaCl, 1% Triton X-100, 0.1% SDS, 2 mM EDTA pH 8.0, 20 mM Tris-HCl pH 8.0) and eluted with 120 μl of elution buffer (1% SDS, 0.1 M NaH_2_CO_3_) and proteinase K (1 μg ml^−1^) at 65 °C overnight to reverse crosslinking. Associated DNA was then purified by extraction using the QIAQuick qPCR kit (Qiagen). qPCR was used to determine recovery of specific DNA fragments (primers in Supplementary Table 1). Within each ChIP experiment, a negative control ChIP was performed using 5 μg of polyclonal rabbit anti-mouse immunoglobulin (IgG).

### Luciferase assays

For transcriptional reporter assays, HUVEC cells were transfected with p75^NTR^ proximal promoter or miR-503 promoter (Switchgear Genomics) constructs using GenJet *In Vitro* DNA Transfection Reagent (SignaGen), and, 24 h later, cells were exposed to L-Glucose or D-Glucose or transduced with *Ad.p75* (*Ad.Null* as control) for 24 h and lysed using buffer supplied with the Dual-Luciferase Reporter Assay System (Promega). Mutated plasmids were generated using GeneTailor Mutagenesis system kit (Invitrogen). To investigate whether miR-503 directly regulates VEGFA, and EFNB2 expression, portions of the 3′UTR of these potentials target genes were inserted downstream of a luciferase open reading frame (pLUC). *VEGFA* 3′UTR (S204537) and *EFNB2* 3′UTR (S213182) vectors were purchased from SwitchGear Genomics. Vectors in which five nucleotide mutations were inserted in the 3′UTR sequences (*VEGFA*: 293–299; *EFNB2*: 1,126–1,132) complementary to the miR-503 ‘seed’ sequence were prepared using the GeneTailor kit (Invitrogen). Primers are listed in Supplementary Table 1. Luciferase constructs were transfected into HEK293T cells together with either pre-miR-503 or a scrambled oligonucleotide sequence (control). Cells were cultured for 48 h and assayed with the Dual-Luciferase Reporter Assay System (Promega).

### Electron microscopy

HUVECs were grown to confluency in an eight-well glass-bottom chamber and were fixed with 2.5% glutaraldehyde in 0.1 M cacodylate buffer and processed for Epon embedding and electron microscopy according to a standard protocol^59^. Transverse sections were made. Isolated particles were also fixed and processed as above. Ultrathin sections were made and analysed using a Tecnai 12 Spirit transmission electron microscope (FEI Co.) equipped with an Eagle 4 k × 4 k charge-coupled device camera.

### Western blot analyses

Western blot was performed as previously described^10^. Nuclear/cytoplasm separation was performed using an NE-PER kit (Pierce). The following antibodies have been used: NF-κBp65 (Millipore, 17–10,060; 1:1,000), EFNB2 (GeneTex, GTX88049; 1:1,000), VEGFA (SantaCruz Biotechnology, sc-152; 1:2,000), MLC2 (Cell Signalling, 3672; 1:1,000), p-MLC2 (Ser19; Cell Signalling, 3675; 1:500), MYPT1 (Cell Signalling, 8574; 1:1,000), p-MYPT1 (Cell Signalling, 4563; 1:500), A/C Laminin (Active Motif, 39287; 1:1,000) and Tubulin (Cell Signalling, 2148; 1:1,000).

### Animal experiments

Mouse experiments are reported in accordance with the Animal Research Report of *In Vivo* Experiments (ARRIVE) guidelines. Experiments were performed in accordance with the Animal (Scientific Procedures) Act (UK) 1986 prepared by the Institute of Laboratory Animal Resources and under the auspices of UK Home Office Project and Personal License. Experiments were approved by the University of Bristol Ethical Review Committee. Six- to seven-week-old-male p75KO (ref. 60; genetic background: C57BL/6J) and WT littermates or CD-1 mice were made diabetic using streptozotocin (STZ; Sigma)^3^ or left normoglycemic after STZ buffer administration alone. STZ was delivered intraperitoneally (i.p.) for five consecutive days (40 mg kg^−1^ in citrate buffer per day). Fourteen days after the first STZ injection, glycaemia at fast and glycosuria were measured and only those mice with glycaemia above 200 mg dl^−1^ and overt glycosuria entered the protocol. Absence of hyperglycaemia and glycosuria in buffer-injected non-diabetic mice was also verified.

Three months after the onset of hyperglycaemia, unilateral hindlimb ischaemia was surgically induced by left femoral artery occlusion. For gene transfer experiment, mice were anaesthetized (tribromoethanol, 880 mmol kg^−1^ i.p., Sigma) to induce limb ischaemia using a refined procedure that consists of ligation (with a 7-0 silk suture) in two points and electrocoagulation of the upper part of the left femoral artery, but leaving the femoral vein and nerve untouched. Immediately after, *Ad.decoy503, Ad.p75, Ad.dnIKK2, Ad.miR-503* or *Ad.Null* (10^9^ plaque-forming unit) was delivered to the ischaemic adductor muscle. The superficial blood flow of the ischaemic and contralateral feet was sequentially analysed (at 30 min, and 7, 14 and 21 days) by colour laser Doppler (Lisca colour laser Doppler, Perimed, Sweden, and Moor, USA), and the ratio of blood flow between the ischaemic foot and the contralateral foot was calculated and used as an index of % blood flow recovery. At 3 and 21 days post-ischaemia, *n*=6 mice per group were killed for molecular biology analyses. At 21 days post-ischaemia, adductor muscles from terminally anaesthetized mice (*n*=6) were *in situ* perfused with heparinased PBS for 1 min and then with 10% buffered formalin for 5 min via a cannula inserted into the abdominal aorta in the direction of limbs. Ischaemic and contralateral muscles were then removed, kept in 4% buffered formalin for 24 h and processed for paraffin embedding^61^.

For analysis of vascularization, muscular sections were stained as previously described^11^. Briefly, sections were incubated overnight at 4 °C with Alexa 488-conjugated isolectinB4 (Molecular Probes, I21411; 1:100) to identify ECs and Cy3-conjugated α-vascular smooth actin (Sigma-Aldrich, C6198; 1:100) to recognize vascular smooth muscle cells (which are part of the arteriole walls). Slides were observed under a fluorescence microscope (Olympus CX41, Olympus). High-power fields were captured (at × 400) and the number of capillaries and arterioles per field were counted. At least 30 randomly chosen fields were evaluated. Arterioles were recognized from venules by their morphology. In fact, arterioles have a lumen that is circular or elliptical and well opened by the perfusion/fixation. Moreover, arterioles have one or more continuous layers of vascular smooth muscle cells in the tunica media. Capillaries can be also identified by this staining as being the small vessels composed by one or two ECs stained by lectin, but missing vascular smooth muscle cells. Capillary and arteriole densities were expressed as number of vessels per mm^2^ of muscular sections.

### Isolation of ECs and pericytes from mouse ischaemic limb muscles

Adductor muscles at 3 days post-ischaemia induction in diabetic and non-diabetic mice were rinsed and digested with collagenase II (Worthington) plus DNase I (Sigma) using gentleMACS Dissociator, following the manufacturer’s protocol. Next, ECs and pericytes were immunomagnetic sorted using CD31 or NG2 antibodies, respectively (Miltenyi Biotech), as reported in ref. 62. Purity of cell preparations was analysed by flow cytometry using CD31-FITC (Miltenyi, 130-102-970, 1:50) and NG2-PE (eBioscience, 8012-6504-120, 1:50) antibodies.

### Permeability assays

Evans Blue (0.5% ; Sigma) was injected into the tail vein and allowed to circulate for 30 min. The mice were then killed, blood was drained and the adductor group muscle was excised and dried at 55 °C. Evans Blue (Sigma) in tissues was extracted with formamide for 24 h at 55 °C, and its fluorescence at 610 nm was measured by a fluorescent reader (Bio-Tek)^63^.

### miRNA *in situ* hybridization

For the detection of miR-503 in mouse hindlimb muscle, sections were rehydrated in histoclear and graded concentrations of ethanol. Slides were then boiled for 10 min within 10 mM sodium citrate pH 6.0, cooled to room temperature (RT), incubated with 10 μg ml^−1^ proteinase K at 37 °C for 20 min and fixed in 4% paraformaldehyde (PFA) for 10 min at RT. Endogenous peroxidases were blocked by incubation with 3% H_2_O_2_ in H_2_O. Then, slides were incubated with hybridization buffer (50% formamide, 4 × SSC, 2.5 × Denhadrt’s solution, 2.5 mg ml^−1^ salmon DNA, 0.6 mg ml^−1^ yeast tRNA, 0.025% SDS and 0.1% blocking reagent) at 60 °C for 1 h followed by a 60-°C overnight incubation with 40 nM miR-503 or scramble miRCURY LNA Detection probe, 5′-DIG labelled (Exiqon) in the same buffer. Melting temperatures were 61 and 78 °C. Immunodetection was performed by blocking the sections in 1% blocking reagent in PBS and 10% normal goat serum for 1 h at RT, followed by an overnight incubation at 4 °C with an anti-DIG-POD antibody (Roche Applied Science) diluted 1:400. Slides were then incubated with TSA-plus fluorescein isothiocyanate for 10 min at RT to detect miR-503. Detection of isolectin-B4 (Molecular Probes, I2141, 1:100) and NG2 (Abcam, ab81104; 1:100) was subsequently performed using AlexaFluor 633 (Molecular Probes, 1:500) or AlexaFluore568 (Molecular Probes, 1:500) secondary antibody.

### Immunohistochemistry

Samples were fixed in 4% formalin, embedded in paraffin wax and sectioned for histological staining. Sections were incubated overnight at 4 °C with p75^NTR^ (Millipore, 07–476; 1:100) and NG2 antibody (Abcam, ab81104; 1:100). Capillary and arteriole densities were determined using fluorescent microscopy on sections stained with Alexa 488-conjugated isolectin-B4 (Molecular Probes, I2141, 1:100 ) and Cy3-conjugated α-vascular smooth actin (Sigma, C6198, 1:200).

### Statistical analysis

Comparisons between different conditions were assessed using two-tailed Student’s *t*-test. If the normality test failed, the Mann–Whitney test was performed. Necrotic toe endopoint was analysed using Cochran–Armitage trend test. Differences among groups were elicited using analysis of variance followed by Bonferroni *post hoc* analyses as appropriate. Continuous data are expressed as mean±s.e.m. of three independent experiments, each performed in triplicate or quintuplicate. *P* value<0.05 was considered statistically significant. Analyses were performed using GraphPad Prism v5.0.

## Additional information

**Accession codes:** miRNA array expression data have been deposited in the NCBI Gene Expression Omnibus database under accession code GSE53899.

**How to cite this article:** Caporali, A. *et al.* p75^NTR^-dependent activation of NF-κB regulates microRNA-503 transcription and pericyte–endothelial crosstalk in diabetes after limb ischaemia. *Nat. Commun.* 6:8024 doi: 10.1038/ncomms9024 (2015).

## Supplementary Material

Supplementary Information Supplementary Figures 1-12 and Supplementary Table 1
