# Supplementary material for: p75NTR-dependent activation of NF-κB regulates microRNA-503 transcription and pericyte–endothelial crosstalk in diabetes after limb ischaemia
Source: Nat Commun. 2015 Aug 13;6:8024. doi: 10.1038/ncomms9024 (PMC4538859; doi:10.1038/ncomms9024)
Supplement: Supplementary Information — Supplementary Figures 1-12 and Supplementary Table 1 [file ncomms9024-s1.pdf]

SUPPLEMENTARY FIGURES

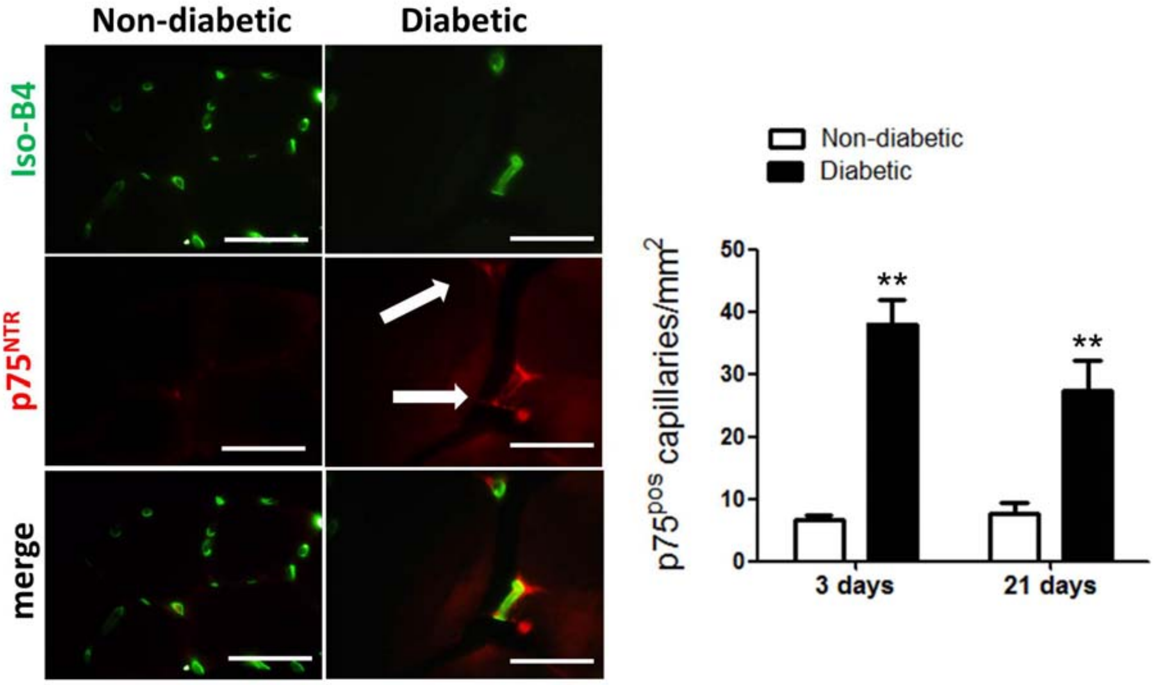

Supplementary Figure 1: Expression of p75^{NTR} in microvascular ECs of limb muscle

A, Representative pictures of ischemic muscular sections from diabetic and non-diabetic mice (n=6/group) stained with an antibody for mouse p75^{NTR} (red fluorescence) and with the Isolectin-B4 (Iso-B4; green fluorescence) at 21 days after ischemia. Capillary EC which are positive for p75^{NTR} express yellow fluorescence (from merging of the red and green fluorescence) and are pointed by arrows. Scale bar: 50μm **B**, Bar graph quantifies the density of capillaries expressing p75^{NTR} in ischemic limb muscles of normoglycemic and diabetic mice. Values are means±SEM (n=6/group). **P<0.01 vs. non-diabetic mice. Unpaired two-tailed Student's t-test was applied when comparing two groups. All values are mean±SEM of three independent experiments.

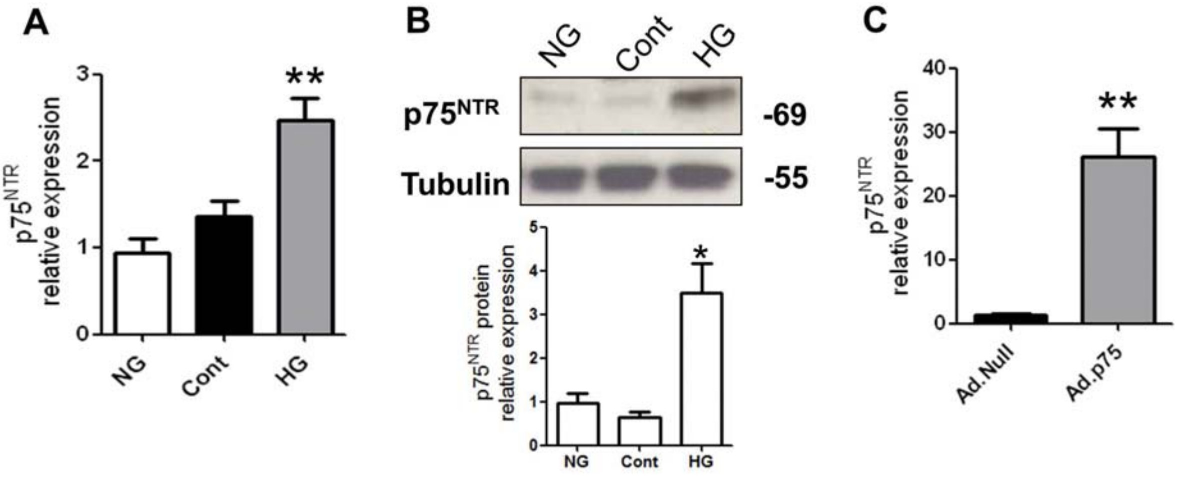

D

Name	logFC	AveExpr	t	P.Value	adj.P.Val	B
hsa-miR-503	1.7783	7.6514	7.3672	1.17E-07	8.80E-05	7.7361
hsa-miR-921	1.0771	7.4448	7.0847	2.27E-07	8.80E-05	7.1135
hsa-miR-198	1.8173	5.8589	6.3758	1.25E-06	0.0003	5.5024
hsa-miR-30c-2*	2.8781	7.8856	6.1012	2.45E-06	0.0005	4.8612
hsa-miR-665	1.9053	7.8637	5.6948	6.77E-06	0.0011	3.8971
miRPlus_42526	3.4152	9.0718	5.5995	8.60E-06	0.0011	3.6689
hsa-miR-187*	2.1065	5.6597	5.3824	1.49E-05	0.0017	3.1462
hsa-miR-371-5p	3.4456	9.6093	5.0274	3.68E-05	0.0032	2.2851
hsa-miR-183*	1.3027	5.8091	5.0194	3.76E-05	0.0032	2.2656
hsa-miR-30b*	2.3325	8.0864	4.8609	5.64E-05	0.0040	1.8793
hsa-miR-483-5p	1.6567	5.5381	4.8567	5.70E-05	0.0040	1.8691
hsa-miR-658	1.7171	7.9106	4.7253	7.99E-05	0.0052	1.5487

Supplementary Figure 2: miR-503 expression in microvascular ECs and miRNA array analysis

A, HMVECs were exposed to high-glucose (HG), cultured in normal glucose (NG) or osmotic control (Cont; L-Glucose) conditions for 24hrs and expression of p75^{NTR} was analyzed by qPCR. **p<0.01 vs NG or Cont (n=3), unpaired two-tailed Student's t-test; **B**, Representative Western blot for p75^{NTR} and tubulin and protein quantification *p<0.05 vs NG or Cont (n=3), unpaired two-tailed Student's t-test **C,** Expression of p75^{NTR} in HUVECs after transduction with *Ad.p75*. Gene expression was normalized to 18S expression. **p<0.01 *vs. Ad.Null* (n=3), unpaired two-tailed Student's t-test; All values are mean±SEM of three independent experiments. **D**, Ranked list of miRNA differentially expressed in HUVECs transduced with *Ad.p75* or *Ad.Null* (as control). miRNAs were ranked by differential expression and statistical significance. The default table of Limma is ranked by B-statistics (B), closely related to the adjusted p-value. The B-statistic used for this analysis is the log-odds that that gene is differentially expressed and it is automatically adjusted for multiple testing by assuming that 1% of the genes are expected to be differentially expressed.

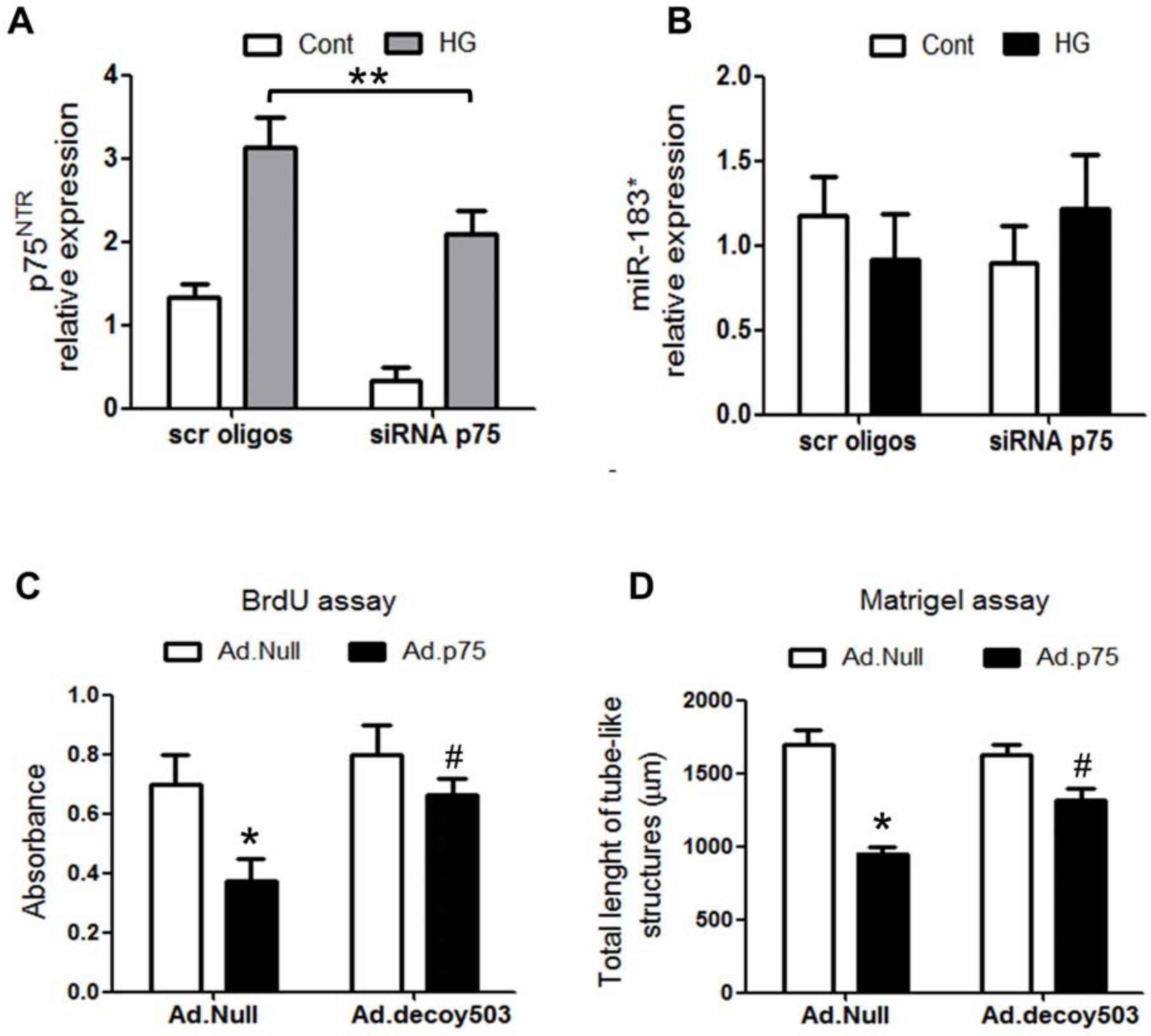

Supplementary Figure 3: The $p75^{NTR}$ receptor regulates EC function
A, $p75^{NTR}$ expression in HUVECs transfected with siRNA for $p75^{NTR}$ or scramble oligos (scr oligos) and treated with HG or L-Glucose (Cont) for 24hrs; $^{**}p<0.01$; two-way ANOVA (n=5); **B,** miR-183* expression in the conditions described above; **E**, Proliferation assay in HUVECs transduced with *Ad.Null*, *Ad.p75* or *Ad.decoy503* adenovirus; **F,** Matrigel assay in the same conditions described above. Results are described as total tube length. For **E** and **F**: $^{*}p<0.05$ *vs*. *Ad.Null*, $^{\#}p<0.05$ *vs. Ad.p75* (n=3), unpaired two-tailed Student's t-test. All values are mean±SEM of three independent experiments.

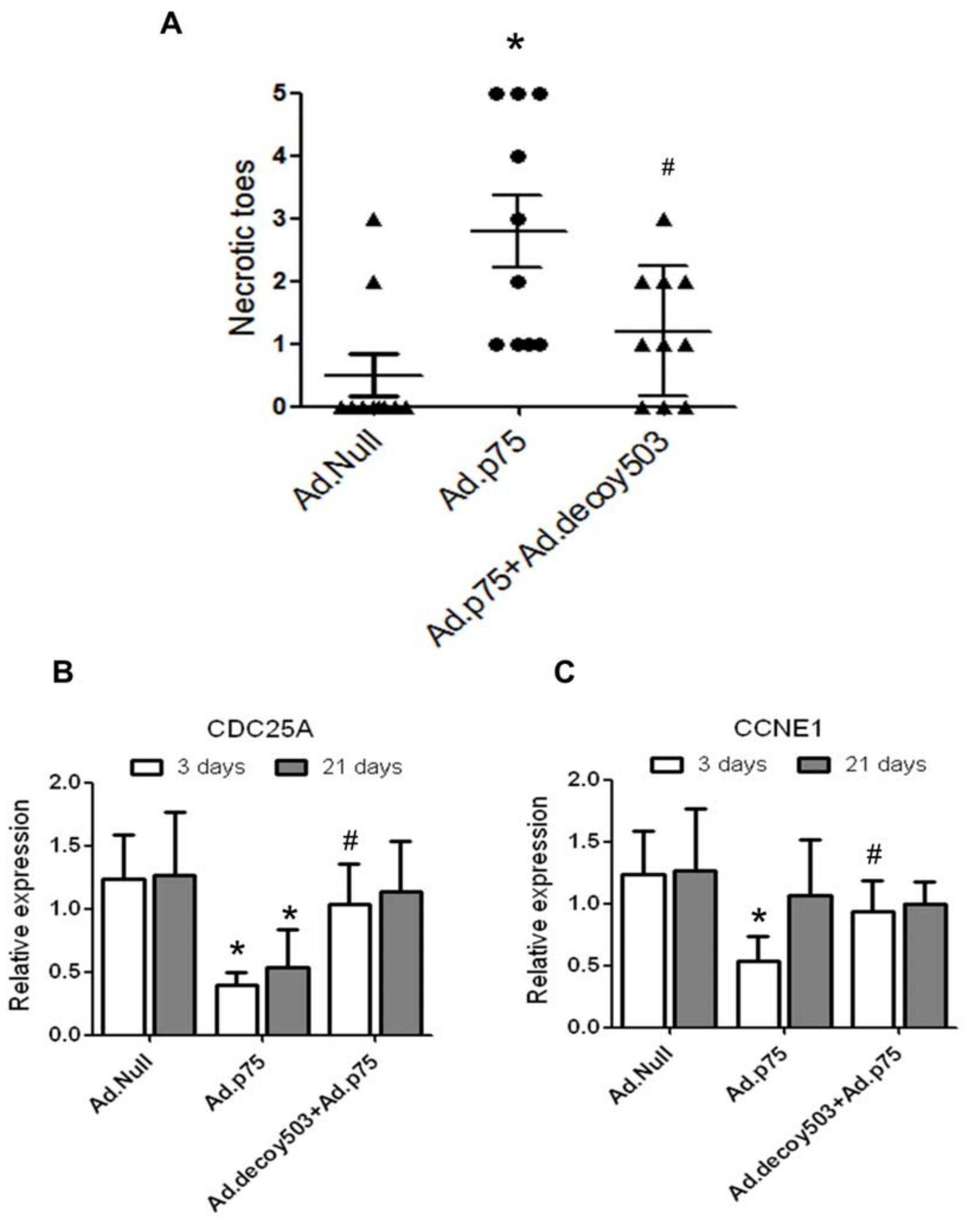

Supplementary Figure 4: Clinical outcomes and target gene expression after p75^{NTR} gene transfer in limb muscles of ischemic mice

A, Analysis of necrotic toes of non-diabetic mice injected with *Ad.p75*, *Ad.Null* or *Ad.p75* and *Ad.decoy503* together in ischemic limb muscles (n=12/group). *p<0.05 vs. Ad.Null, #p<0.05 vs. Ad.p75, necrotic toes endopoint was analyzed using Cochran-Armitage trend test **B,** Relative expression of *CDC25A* and *CCNE1* in ischemic adductors of non-diabetic mice given *Ad.p75*, *Ad.Null* or *Ad.p75* and *Ad.decoy503* together (n=6/group). *p<0.05 *vs. Ad.Null*, $^{\#}$p<0.05 *vs. Ad.p75*, Mann–Whitney nonparametric test was applied. All values are mean±SEM of three independent experiments.

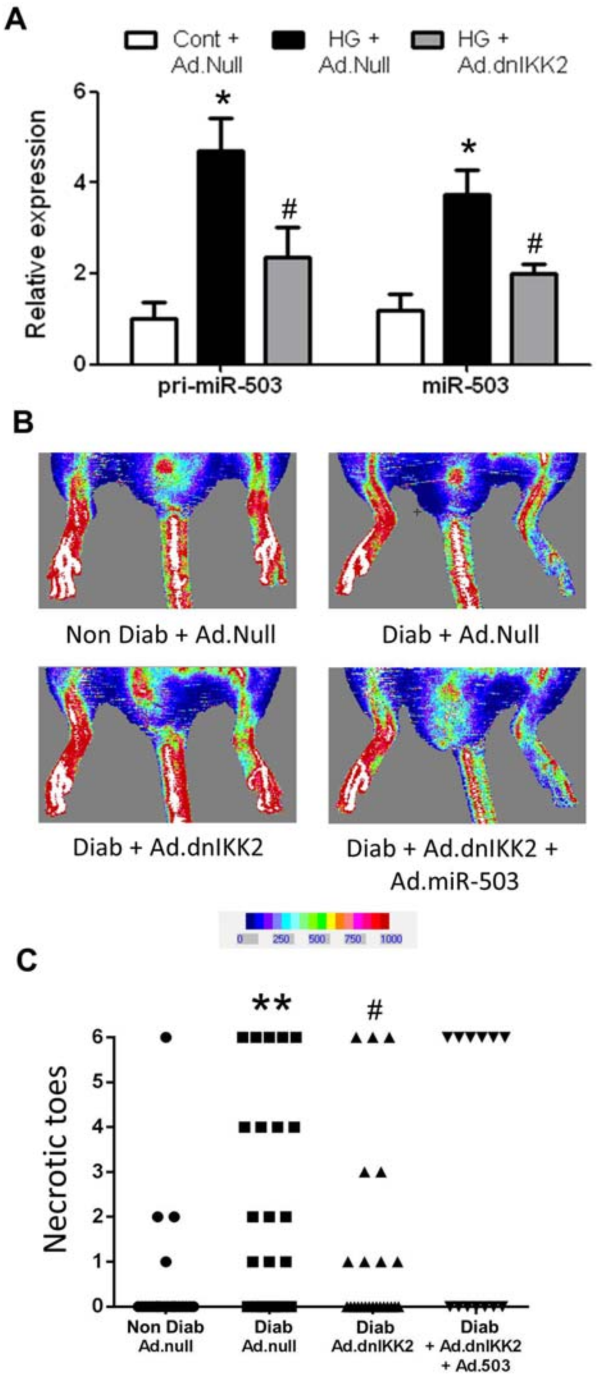

Supplementary Figure 5: miR-503 regulation by NF-kB and clinical outcomes after dnIKK2 gene transfer in limb muscles of diabetic and ischemic mice

A, HUVECs were transduced with *Ad.Null* or *Ad.dnIKK2* and treated with HG or osmotic control (L-Glucose). qPCR was carried out to measure the expression of pri-miR-503 and mature miR-503; *p<0.05 vs Cont+*Ad.Null*; $^{\#}$p<0.05 vs HG+*Ad.Null* (n=3), unpaired two-tailed Student's t-test All values are mean±SEM of three independent experiments. **B,** Representative color laser Doppler images are taken at 21 days post-ischemia. **C,** Analysis of necrotic toes of non-diabetic mice and diabetic mice injected with *Ad.dnIKK2*, *Ad.miR-503* or *Ad.Null* in ischemic limb muscles (n=12/group). **p<0.01 *vs.* Non Diab+*Ad.Null*; $^{\#}$p<0.05 *vs.* Diab+*Ad.Null*, necrotic toes endopoint was analysed using Cochran-Armitage trend test. Data show individual data points, where a designation of "6" indicates foot necrosis or impaired foot locomotion.

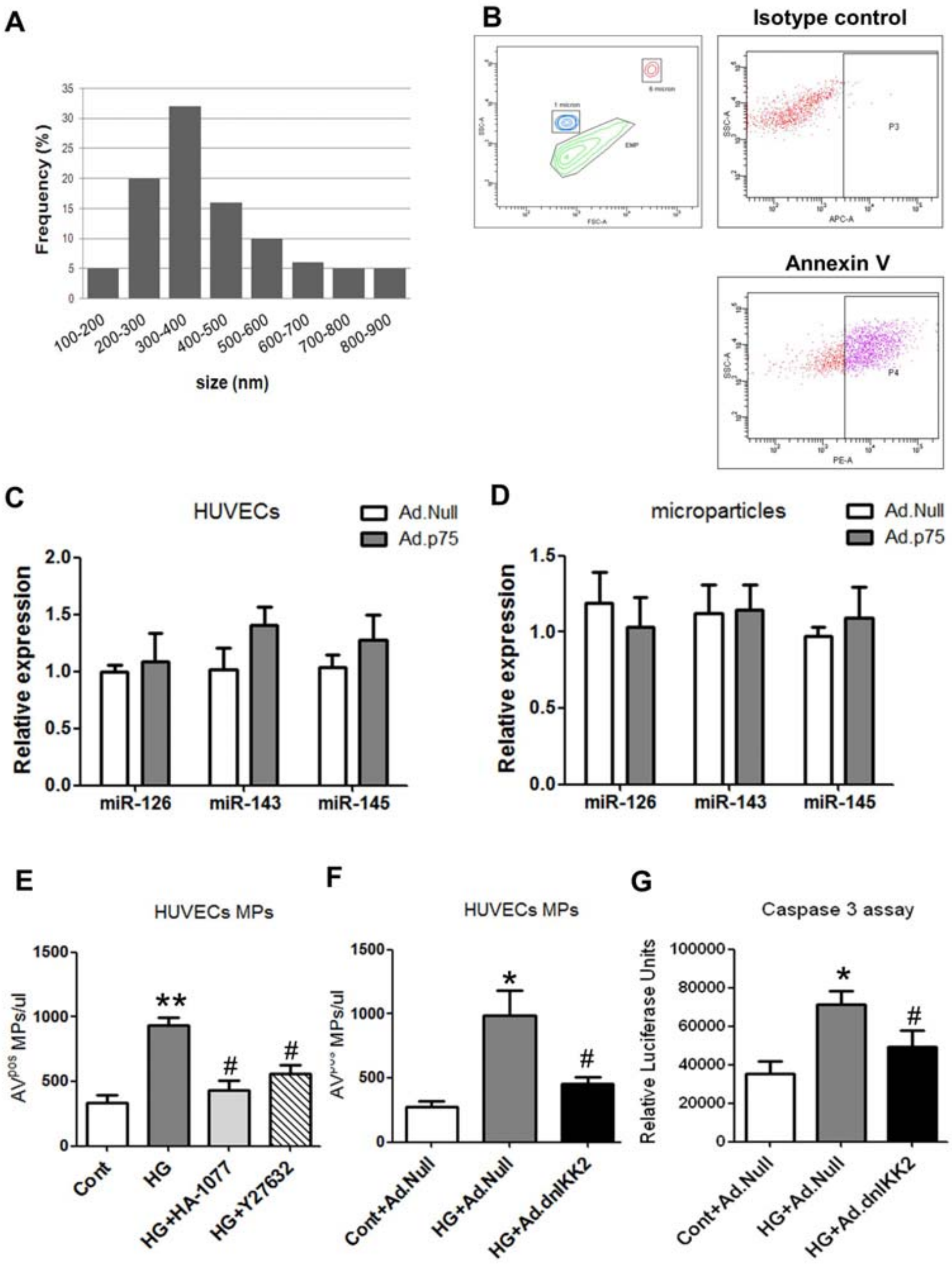

Supplementary Figure 6: Endothelial microparticles analysis

A, Size distribution of isolated MPs from p75^{NTR}-transduced HUVECs (n=500 cells). **B**, Flow cytometric analysis of MPs from p75^{NTR}-transduced HUVECs. p75^{NTR} increased the release of HUVEC-derived MPs expressing AnnexinV (AnnV^{Pos}) on the MP-surface; **C**, Expression of miR-126, miR-143 and miR-145 in HUVECs and in **D**, isolated MPs from medium of p75^{NTR}-transduced HUVECs. **E,** HUVECs were cultured in HG and osmotic control medium (Cont) and treated with Rho kinase inhibitors Y27632 (10µM) or HA-1077 (10µM) and MPs were collected from the medium by centrifugation and analyzed for Annexin V by flow cytometry. **p<0.01 vs Cont; #p<0.05 vs HG (n=3), unpaired two-tailed Student's t-test. **F,** HUVECs were cultured in same conditions described above and transduced with the *Ad.Null* or *Ad.dnIKK2*. **G**, Caspase-3 activity in HUVECs treated as described above, For **F** and **E**: *p<0.05 vs Cont/*Ad.Null*; $^{\#}$p<0.05 vs HG/*Ad.Null* (n=3), unpaired two-tailed Student's t-test. All values are mean±SEM of three independent experiments.

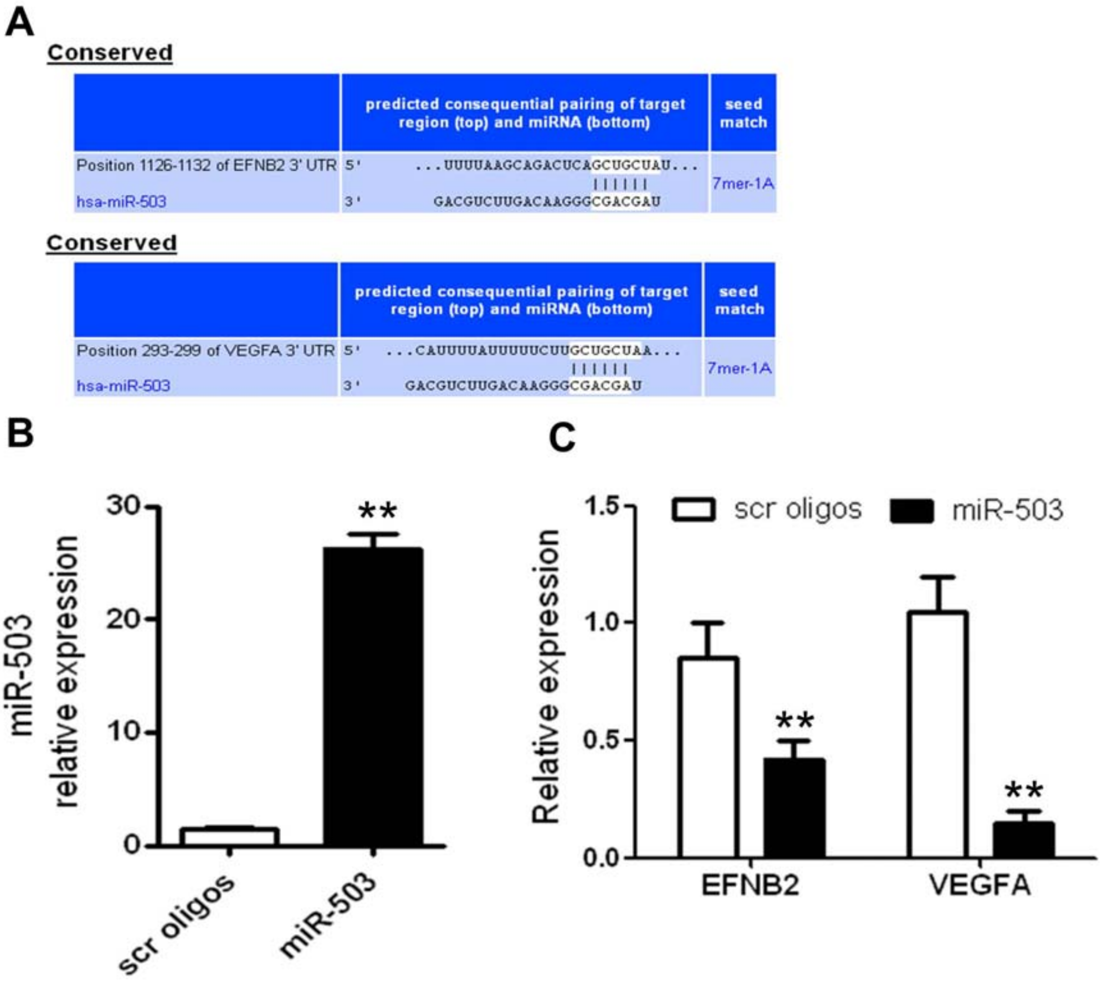

Supplementary Figure 7: miR-503 targets EFNB2 and VEGFA
A, Seed sequences in 3'UTR of *EFNB2* and *VEGFA* from Targetscan 6.2; **B**, miR-503 expression in pericytes 48hrs after miR-503 or control oligos transfection; **C**, Expression of *VEGFA* and *EFNB2* in pericytes 48hrs after transfection with siRNA for *EFNB2* and *VEGFA*; **p<0.01 *vs.* scr oligos (n=3), unpaired two-tailed Student's t-test. All values are mean±SEM of three independent experiments.

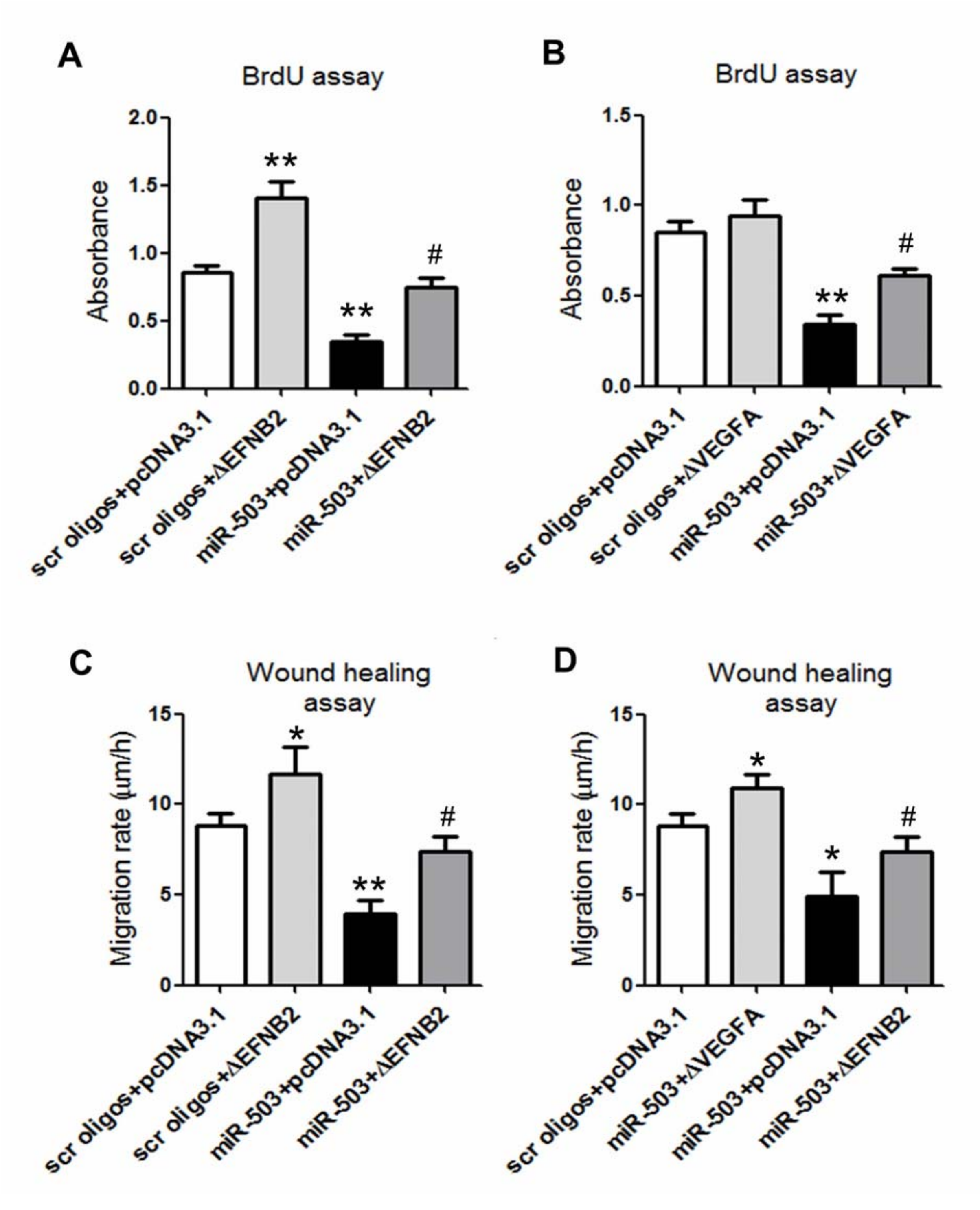

Supplementary Figure 8: Overxpression of 3'UTR-mutated target gene restores proliferation and migration in pericytes overexpressing miR-503

HUVECs expressing either Δ*EFNB2* or Δ*VEGFA* or vector alone (pcDNA3.1) were transfected with mature miR-503 or a scramble sequence and further cultivated for 24 hrs. Then, proliferation (**A** and **B**) or migration (**C** and **D**) were assayed. Δ*EFNB2* or Δ*VEGFA* partially restored the proliferation (A and B, respectively) and migration (C and D, respectively) in HUVEC transfected with pre-miR-503. $^{**}p<0.01$ or $^{*}p<0.05$ *vs.* scr oligos+pcDNA3.1; $^{\#}p<0.05$ *vs.* miR-503+ pcDNA3.1 (n=5), unpaired two-tailed Student's t-test. All values are mean±SEM of three independent experiments.

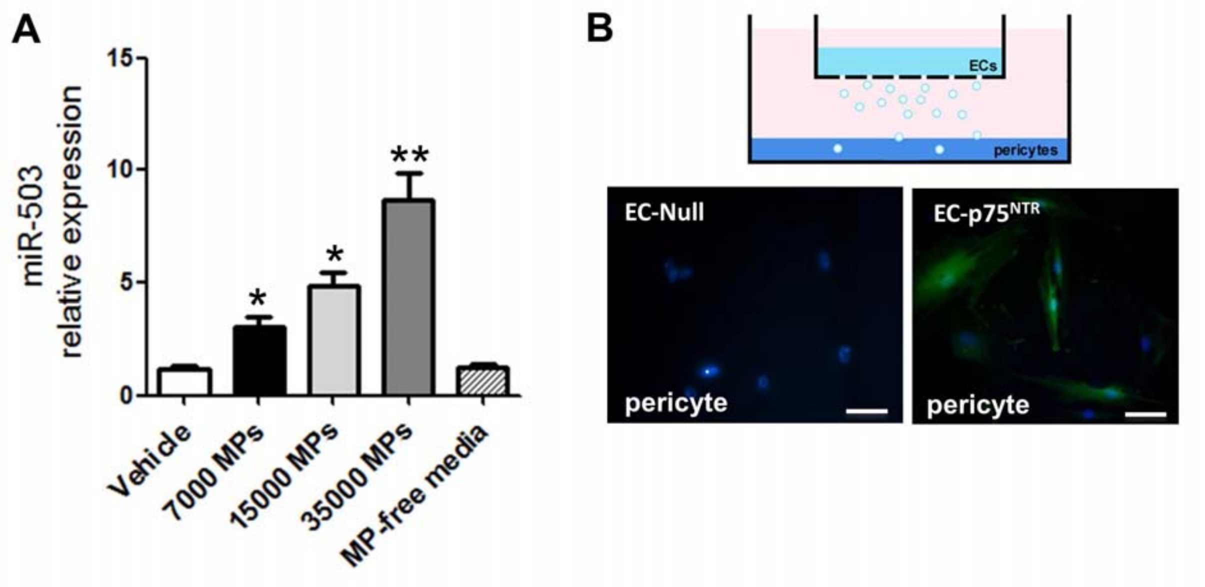

Supplementary Figure 9: The uptake of MPs by pericytes
A, pericytes incubated for 24h with MPs-miR-503 increased significantly the intracellular levels of miR-503 in dose-dependent manner. *p<0.05, **p<0.01 vs. vehicle` (n=5), unpaired two-tailed Student's t-test. All values are mean±SEM of three independent experiments. **B,** An *in vitro* co-culture system of HUVECs (top compartment) with pericytes (bottom compartment) in which the cells are separated by a membrane to prevent direct cell contact has been setup HUVECs were labeled with DiO (green fluorescence) and transduced with *p75*^{NTR}-overexpressing adenovirus or *Null*-control adenovirus and fluorescence was analyzed in pericytes after 48hrs of co-culture by fluorescent microscopy; Scale bar 50 μm.

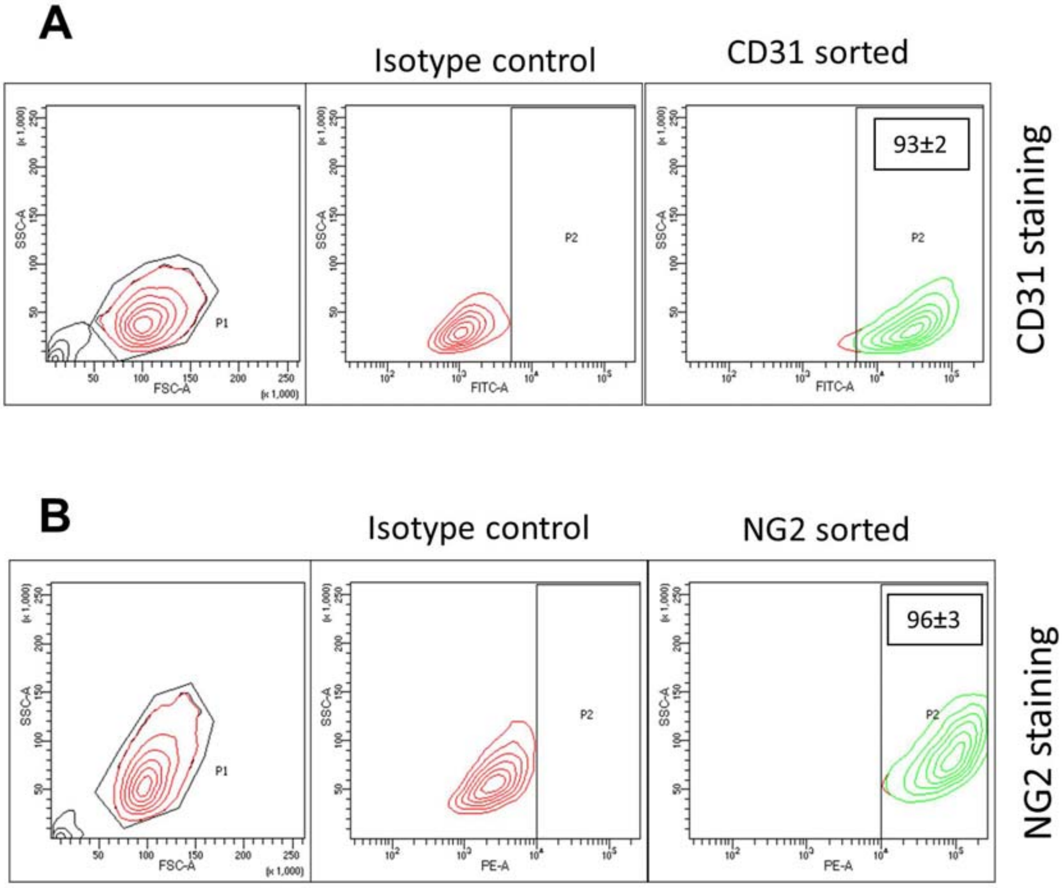

Supplementary Figure 10: Flow cytometry analysis of CD31 and NG2 sorted cells from limb muscle

Cells were isolated using magnetic beads conjugated with CD31 and NG2 antibodies. Sorted cells were stained using **A**, CD31-FITC or **B**, NG2-PE conjugated antibody. Boxed: percentage of CD31 and NG2 in sorted cells (n=3); All values are mean±SEM of three independent experiments.

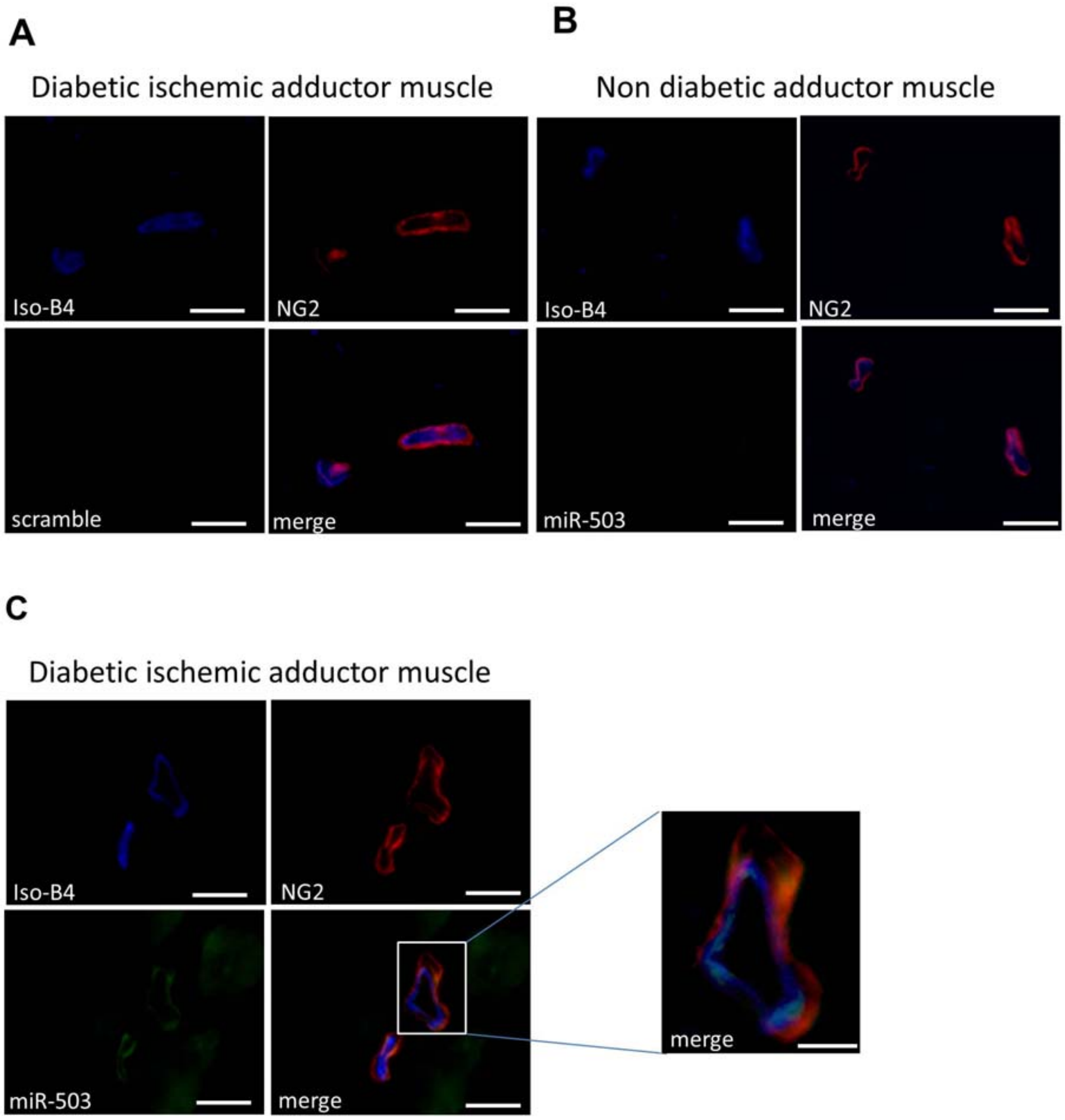

Supplementary Figure 11: miR-503 *in situ* hybridization
Localization of miR-503 (green fluorescence) by *in situ* hybridization in endothelial cells (Isolectin-B4 blue fluorescence) and pericytes (NG2, red fluorescence) in the ischemic limb muscles of diabetic and non-diabetic mice. **A**, Ischemic adductor muscle of diabetic mice hybridized with scrambled sequence probes as negative control (n=6). **B,** Non diabetic adductor muscle hybridized with miR-503 probe (n=6). **C**, Ischemic adductor muscle of diabetic mice hybridized with miR-503 probe (n=6). Scale bars 50 µm. Insert: Higher magnification image of small vessel in ischemic adductor muscles of diabetic mice. Scale bars 10µm.

Full unedited gel for Figure 1

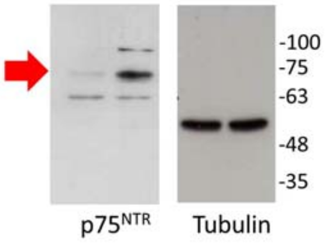

Full unedited gel for Figure 3

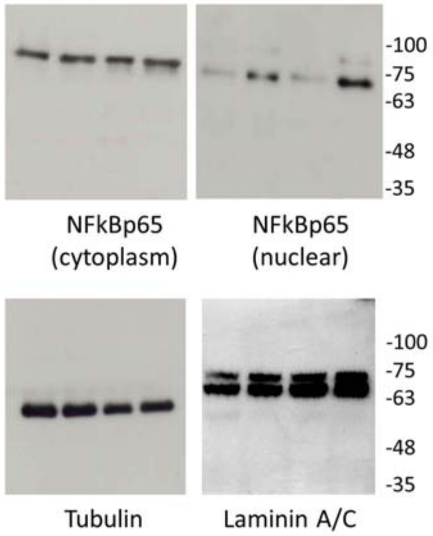

Full unedited gel for Figure 5

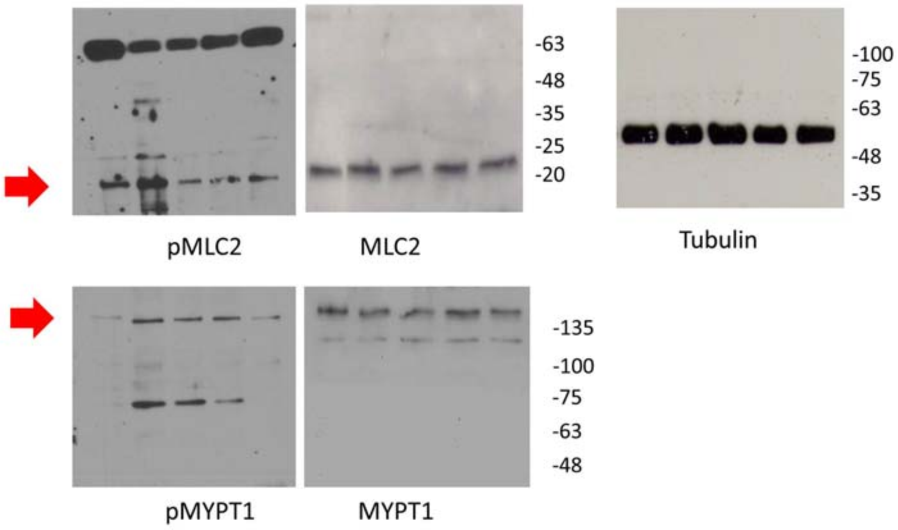

Full unedited gel for Figure 6

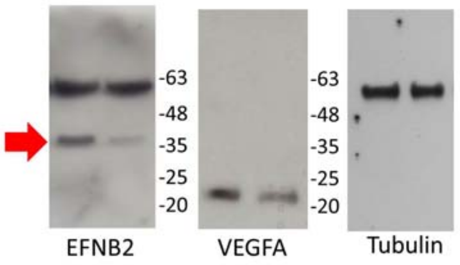

Full unedited gel for Suppl. Figure 1

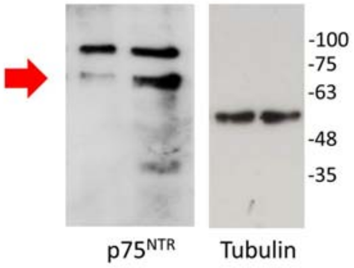

Supplementary Figure 12: Original data for immunoblot related to main Figures

SUPPLEMENTARY TABLES

Table 1 - List of primers used in the study

	**Forward**	**Reverse**
mut 3'UTR EFNB2	5'ATGATTTTTTAAGCAGACTCAATTTAA TATACTTATCA-3'	5'TGAGTCTGCTTAAAAAATCATC CAAAGCAGA-3'
mut 3' VEGFA	5'ATTCGCCATTTTATTTTTCTATTTAATT AATCACCGAG-3'	5'AGAAAATAAAATGGCGAATCCA ATTCCAA-3'
**ChIP primers**		
NF-kBp65	5'-CTTCCTTCCTCCCTGTCTCC-3'	5'-GTTTTTCTGCCTGCCGTAAC-3'
IkBα	Millipore primer set 17-10060	
